# Analysis of Italian isolates of *Pantoea stewartii* subsp. *stewartii* and development of a real-time PCR-based diagnostic method

**DOI:** 10.3389/fmicb.2023.1129229

**Published:** 2023-04-27

**Authors:** Valeria Scala, Luigi Faino, Francesca Costantini, Valeria Crosara, Alessio Albanese, Nicoletta Pucci, Massimo Reverberi, Stefania Loreti

**Affiliations:** ^1^Research Centre for Plant Protection and Certification, Council for Agricultural Research and the Analysis of Agricultural Economics (CREA), Rome, Italy; ^2^Department of Environmental Biology, Sapienza University of Rome, Rome, Italy

**Keywords:** *Pantoea stewartii* subsp. *stewartii*, plant pathogens, diagnostic methods, genome sequencing, regulated pest, phylogeny

## Abstract

*Pantoea stewartii* subsp. *stewartii* (Pss) causes Stewart’s vascular wilt of maize, and it is responsible for serious crop losses. Pss is indigenous to North America and spreads with maize seeds. The presence of Pss has been notified in Italy since 2015. The risk assessment of the entry of Pss in the EU from the United States through seed trade is in the order of magnitude of hundred introductions per year. Several molecular or serological tests were developed for the detection of Pss and used as official analysis for the certification of commercial seeds. However, some of these tests lack adequate specificity, not allowing to correctly discriminate Pss from *P. stewartii* subsp. *indologenes* (Psi). Psi is occasionally present in maize seeds and is avirulent for maize. In this study, several Italian isolates of Pss recovered in 2015 and 2018 have been characterized by molecular, biochemical, and pathogenicity tests; moreover, their genomes have been assembled through MinION and Illumina–sequencing procedures. Genomic analysis reveals multiple introgression events. Exploiting these results, a new primer combination has been defined and verified by real-time PCR, allowing the development of a specific molecular test able to detect the presence of Pss down to the concentration of 10^3^  CFU/ml in spiked samples of maize seed extracts. Due to the high analytical sensitivity and specificity achieved with this test, the detection of Pss has been improved disentangling the inconclusive results in Pss maize seed diagnosis, overcoming its misidentification in place of Psi. Altogether, this test addresses the critical issue associated with maize seeds imported from regions where Stewart’s disease is endemic.

## 1. Introduction

To date, approximately 30% of food loss is caused by plant pathogens, and a lot of efforts are being made to reduce this percentage. Globalization has an impact on plant disease management (Jeger et al., 2021; Muluneh, 2021). The risk of new diseases caused by non-endemic pathogens is of particular concern because endemic plants do not evolve any resistance gene to control the invasive pathogen. Several pathogens confined to specific regions are, therefore, listed in the FAO-ICPP (International Plant Protection Convention) and EPPO documents. These pathogens are under strict surveillance and specific protocols are used for their detection. Sometimes, the detection methods are not specific enough to distinguish among different pathogen species or subspecies.

In the frame of EUROPHYT (European Union Notification System for Plant Health Interceptions), the Plant Protection Services carried out the surveillance of the Italian territory and notified the presence of *Pantoea stewartii* subsp. *stewartii* (Pss), the causal agent of Stewart’s wilt disease of *Zea mays* in 2015, 2016, 2017, 2018, 2020, and 2022 in the Emilia-Romagna, Friuli Venezia Giulia, Veneto, and Lombardia regions. In Europe, Stewart’s wilt was randomly reported but has not become permanently established.

*Pantoea stewartii* subsp. *stewartii* is a gram-negative bacterium, taxonomically classified as a member of the *Erwiniaceae* family (Adeolu et al., 2016; Kini et al., 2021) and *Pantoea* genus, together with *P. ananatis*, *P. indologenes*, *P. allii*, and *P. agglomerans*. Pss is listed in the quarantine pests A2 list of the European Plant Protection Organization (EPPO), and it is indigenous to North America and has spread worldwide through maize kernels trading (European Plant and Protection Organization, 2016). Pss is responsible for serious crop losses (Roper, 2011). In the north-central and eastern areas of the United States of America, the economic impact of this disease is minimal for the presence of resistant cultivars and the use of systemic seed-applied insecticides, whereas the susceptible varieties are severely affected and may be destroyed at the seedling stage (Coplin et al., 2002). The disease symptoms are grouped into two major phases related to two major cycles of infection as follows: (i) wilt and (ii) leaf blight (Roper, 2011). The wilt phase occurs when young seedlings are infected with Pss. Water-soaked lesions appear on the young leaves and seedlings become severely wilted. The plants usually die when infected at the seedling stage. The leaf blight symptoms occur when mature plants are infected. The leaf blight phase is most apparent after tasseling and does not generally cause the death of the plant, resulting primarily in vascular chlorosis and necrosis with little wilting. In addition, the bacteria can sometimes spread out of the xylem causing pith rot in mature sweet maize plants. In resistant varieties, lesions are usually limited to few centimeters depending on the level of resistance (Pataky and Headrick, 1988; Claflin, 2000; Freeman and Pataky, 2001). When the pathogen grows to high cell densities, it switches to a biofilm stage invading systemically the host tissue and producing an exopolysaccharide, called ‘stewartan’. The stewartan can occlude the xylem vessel leading to wilting and causing plant death (Dolph et al., 1988; Leigh and Coplin, 1992; Beck von Bodman and Farrand, 1995). Pss is found in different plant tissues such as roots, stalks, leaf blades and sheaths, tassels, cobs, husks, and kernels and penetrates the seed deeply but not the embryo (Pataky and Ikin, 2003; Roper, 2011). It is worthy of mention that asymptomatic infection in maize plants with Pss is not known to occur (Coplin et al., 2002).

*Pantoea stewartii* subsp. *stewartii* is vectored in the Americas by *Chaetocnema pulicaria* Melsheimer. This insect is the main overwintering site of the bacterium. In spring, the beetle feeds on the maize plant by introducing bacterial cells into the host through scratching wounds (Pataky and Ikin, 2003). Nevertheless, Pss wilt disease is reported in numerous countries (e.g., Austria, Italy, Slovenia, Canada, Mexico, Russia, and China; CABI, 2018; European Plant and Protection Organization, 2018), and the absence of suitable insect vector prevents its spread (Pal et al., 2019). Stewart’s wilt is a major issue when seeds are destined to be exported and, in relation to this, many countries impose phytosanitary restrictions and require phytosanitary certification stating that seeds are “Pss-free.” Several interceptions of Pss in *Zea mays* are reported from 1999 to 2018, from Hungary, Romania Austria, France, Germany, and the Netherlands (EFSA PLH Panel, 2018).

Several diagnostic methods, based on molecular or serological methodologies, are available for the detection of Pss (e.g., Coplin et al., 2002; Tambong et al., 2008; Wensing et al., 2010; Thapa et al., 2012; Gehring et al., 2014; Tambong, 2015; Pal et al., 2019). Some of these tests are used for commercial seed certification and official analysis (European Plant and Protection Organization, 2016), but false-positive cross-reactions can occasionally mis-identify *P. stewartii* subsp. *indologenes* (Psi). As originally described by Mergaert et al. (1993), Psi can cause symptoms on foxtail millet (*Setaria italica*), pearl millet (*Pennisetum americanum*), leaf blight on rice (Azizi et al., 2019) and on *Dracaena sanderiana* (Zhang et al., 2020), and the rot of pineapple (*Ananas comosus*) and onion (Stumpf et al., 2018), but it is avirulent on maize and is not a “regulated” pest worldwide. Psi is occasionally present in maize seeds of tropical or subtropical origin (Tambong, 2015). Only two tests were developed to discriminate Pss from Psi: a conventional PCR reported by Gehring et al. (2014), Nechwatal et al. (2018), Dreo (2020), and Thapa et al. (2012), and the real-time PCR developed by Pal et al. (2019). The latter allows the detection of Pss directly from maize seeds with acceptable parameters for specificity and sensitivity, without the need for bacterial isolation (Dreo, 2020).

Since the importance of Stewart’s wilt, the efforts to validate and harmonize diagnostic tests for Pss were handled within the EU project Valitest (grant agreement N° 773,139), whose final aim was to improve and harmonize the diagnostic tests for plant health.

The complete genome assembly of *Pss* DC283 was published by Duong et al. (2017) and De Maayer et al. (2017). They reported on a comparative genomic study by using bioinformatic tools to analyze the genomes of 10 *P. stewartii* and 19 *Pantoea ananatis* strains. These studies showed that *P. ananatis* is ubiquitously found in the environment and causes disease in a wide range of plant hosts, whereas Pss is host specific. The genomic differences identified in those studies allowed De Maayer et al. (2017) to postulate on the evolutionary histories of *P. ananatis* and *P. stewartii* strains underline their ecological success and hosts’ range.

Illumina sequencing platforms have enabled widespread bacterial whole genome sequencing, and the data are appropriate for many analyzes. However, the short read length limits its ability to resolve the genomic structure and to track the spread of mobile genetic elements, e.g., those carrying the determinants of antimicrobial resistance (Wick et al., 2017). This issue is resolvable by bacterial genome sequencing through long reads such as those generated by Oxford Nanopore Technologies (ONT) platforms. Loman et al. (2015) showed that long-read data from the Oxford Nanopore MinION can be used to assemble complete bacterial genomes to give an accurate reconstruction of gene order and orientation.

In this study, the bacterial isolates recovered in Italy in 2015 and 2018 are characterized by molecular, biochemical, and pathogenicity tests (European Plant and Protection Organization, 2016), and their whole genome has been sequenced by Oxford Nanopore MinION and Illumina. The following issues have been addressed: (i) the sequencing and assembling of Pss strains with Nanopore MinION; (ii) the phylogeny of the tested isolates with respect to the available data; (iii) the individuation, *in silico*, of a sequence that can discriminate Pss from Psi; and (iv) the development of a real-time PCR test to be used for seed analysis. This study allows us to individuate, at the genomic level, the phylogenetic clade of Pss strains, isolated from maize kernels in Italy in 2015 and 2018, which leads to a novel molecular diagnostic test with improved analytical sensitivity and specificity. The exploit test permits the detection of Pss directly from maize kernels avoiding a previous isolation step and excluding possible false positives resulting when the Psi is present in the seeds.

## 2. Materials and methods

### 2.1. Bacterial strains

All bacteria strains (Table 1) have been cultured on nutrient agar 0.25% d-glucose (NAG) or King’s B medium (KB) (European Plant and Protection Organization, 2016) for 48 h at 27–28˚C. The lyophilized strains are revived and cultured in NAG. Bacterial suspensions have been prepared in phosphate buffer (PB 50 mM, pH = 7) for spiking seed samples and Luria–Bertani medium (LB) (24 h at 28˚C) for bacterial genomic DNA extraction. The concentrations are spectrophotometrically (DS-11 Fx+, Spectrophotometer-Fluorometer Denovix Inc., Wilmington, DE, United States) measured at OD660 = 0.05 corresponding to approximately 10^8^ colony forming units (CFU)/mL. The number of CFU has been determined by plating 100 μl of bacterial suspensions on KB medium, incubated for 48 h at 27–28°C, and the colony counting determined after 2 days.

**Table 1:** Strains used in this study.

Species name	CREA-DC code	Bacterial strain original name	Origin (source if known)
*Pantoea stewartii* subsp. s*tewartii*	CREA-DC 1775	IPV-BO 2766	Italy
	CREA-DC 1869	34,596,1/15^a^	Italy
	CREA-DC 1870	34,258,2/15^a^	Italy
	CREA-DC 1899	49,474/1 (148/18)^a^	Italy
	CREA-DC 1900	49,472/2 (149/18)^a^	Italy
*Pantoea stewartii* subsp. i*ndologenes*	CREA-DC 1923	LMG 2671 NCPPB 1845	Unknown
	CREA-DC 1924	LMG 2630 NCPPB 1877	Hawaii
*Pantoea agglomerans*	CREA-DC 1235	ISF 438	Italy
	CREA-DC 1939	Isolated by CREA-DC	Italy
	CREA-DC 2057	CFBP 6915/IBSBF 1045 ICMP 12205	Brazil
*Pantoea ananantis* subsp. *ananatis*	CREA-DC 2059	CFBP 466/NCPPB 441	Hawaii
	CREA-DC 2060	CFBP 3612/ICMP 1850/NCPPB 1846	Brazil
Sweet maize endophytes	CREA-DC 1868	isolated by CREA-DC	Italy
	CREA-DC 1875	3,156,17^b^	Italy
	CREA-DC 1876	3,157,17^b^	Italy
	CREA-DC 1877	3,154,17^b^	Italy
*Pantoea* spp.	CREA-DC 1925	844,1*	France
	CREA-DC 1926	844,2 *	France
	CREA-DC 1927	619 *	Mexico
	CREA-DC 1928	LNPV 8,14 *	Unknown
	CREA-DC 1929	LNPV 8,15 *	Unknown
	CREA-DC 1930	LNPV 3,37 *	Unknown
	CREA-DC 1931	LNPV 3,55 *	Unknown
	CREA-DC 1932	LNPV 5,74 *	Unknown
*Acidovorax* spp.	CREA-DC 1852	isolated by CREA-DC	Italy
*Brenneria nigrifluens*	CREA-DC 1830	isolated by CREA-DC	Italy
*Brenneria populi*	CREA-DC 1313	NCPPB 4299^T^	Spain
*Clavibacter michiganensis* subsp*. michiganensis*	CREA-DC 1044	isolated by CREA-DC	Italy
*Clavibacter michiganensis* subsp. *sepedonicus*	CREA-DC 1041	NCCPB 2140	Czech Republic
*Erwinia amylovora*	CREA-DC 1219	NCPPB 595	UK
	CREA-DC 1218	NCPPB 683 T	UK
*Pseudomonas syringae* pv. *tomato*	CREA-DC 1364	DC3000	UK
	CREA-DC 1082	NCPPB 2563	UK
*Pseudomonas syringae* pv. *actinidiae*	CREA-DC 1625		Italy
*Pectobacterium carotovorum*	CREA-DC 1249	isolated by CREA-DC	Italy
*Pectobacterium carotovorum* subsp. *atroseptica*	CREA-DC 1156	NCPPB 549	UK
*Xanthomonas arboricola* pv. *juglandis*	CREA-DC 1012	NCPPB 362	UK
*Xanthomonas arboricola* pv. *pruni*	CREA-DC 1151		Italy
*Xanthomonas campestris* pv. *campestris*	CREA-DC 1032		Italy
*Xanthomonas vesicatoria*	CREA-DC 1855	NCPPB 422	Italy
*Xanthomonas gardneri*	CREA-DC 1856	NCPPB 881	Italy
*Xanthomonas euvesicatoria*	CREA-DC 1857	NCPPB 2968	Italy
*Xanthomonas perforans*	CREA-DC 1858	NCPPB 4321	Italy
*Xanthomonas campestris* pv. *pelargoni*	CREA-DC 1214		Italy
*Xylella fastidiosa* subsp. *multiplex*	CREA-DC 2094	CFBP 8416	Italy

Collection of the bacteriology department of the Plant protection, defense and certification research center (CREA-DC code). Istituto Patologia Vegetale Bologna (IPV-BO). Plant Protection Service Emilia-Romagna, Italy (^a^). Plant Protection Service Veneto, Italy (^b^). Laboratory of Microbiology, Department of Biochemistry and Microbiology, Faculty of Sciences of Ghent University Laboratory of Microbiology, Department of Biochemistry and Microbiology, Faculty of Sciences of Ghent University collection Belgian Coordinated Collections of Microorganism (LMG/BCCM). National Collection of Plant Pathogenic Bacteria, York, UK (NCPPB). Istituto Sperimentale Frutticoltura, Italia (ISF). French Collection for Plant Associated Bacteria (CFBP). Biological Institute Culture Collection of Phytopathogenic Bacteria (IBSBF). International Collection of Microorganisms from Plants (ICMP). Kindly provided by ANSES within the EUPHRESCO_A-275 project (*). American Type Culture Collection (DC3000). Type strain (^T^).

### 2.2. Samples preparation and DNA extraction

The samples employed in the real-time PCR analysis consist of different types as follows: (1) 10-fold serial dilutions (from 10 fg to 10 ng/real-time PCR reaction) of genomic bacterial DNA of Pss strain IPV-BO 2766; (2) bacterial genomic DNA extracted from bacterial suspensions at different concentrations (CFU/mL) (i.e., 10^6^ CFU/ml for analytical specificity of all bacterial strains reported in Table 1; from 10^8^ CFU/ml to 10 CFU/ml for analytical sensitivity of Pss strain IPV-BO 2766; (3) spiked samples have been prepared by adding bacterial suspensions from 10^8^ to 10 CFU/ml of Pss strain IPV-BO 2766 to healthy seed extracts (European Plant and Protection Organization, 2016). For samples of types (1) and (2), genomic DNA has been extracted from 1 ml of bacterial cultures using Gentra Puregene Yeast/Bact Kit (Qiagen, Venlo, the Netherlands). For samples of type (3), the DNA of spiked samples is extracted with the DNeasy Plant Mini kit (Qiagen, Venlo, the Netherlands). A negative isolation control (NIC) has been added for each DNA extraction. The DNA concentration is evaluated by Qubit (dsDNA HS Assay kit, Invitrogen, Waltham, MA, United States). The DNA was stored ≤ − 15^◦^C until analysis.

### 2.3. Sequencing by Oxford Nanopore Technologies

Sequence data for the isolates reported in Table 2 has been generated using Oxford Nanopore Technologies (ONT) MinION platform. Nanopore sequencing libraries are prepared according to the manufacturer’s instructions for either SQK-RBK004 or SQK-LSK109 for direct DNA sequencing on an R9.4.1 flowcell or FLO-FLG001 on a MinION device from Oxford Nanopore Technologies (Cambridge, United Kingdom). In brief, for kit SQK-RBK004, approximately ~400 ng of DNA has been purified using AMPure beads, ligated to the indexing adapter, combined in one sample, and subsequently ligated to the RAP adapter prior to sequencing. DNA samples have been run on the Nanopore flowcell version 9.4.1 until pore life ended. Guppy basecalling has been performed on a GPU card Nvidia GTX 1070 8 Gb. For samples 1869 e 1870, a single Flongle flow cell has been used for each strain in combination with the SQK-LSK109. MinKNOW software (v22.08.9) together with Guppy (v6.2.12) is used to demultiplex and basecall the data.

**Table 2:** Isolates sequenced using Oxford Nanopore Technologies (ONT) MinION platform.

Strain number*	Species name	Source	Year
CREA-DC 1235	*Pantoea agglomerans*	*Prunus armeniaca*	
CREA-DC 1775	*Pantoea stewartii* subsp. *stewartii*	*Zea mays*	Before 2000
CREA-DC 1788	*Pantoea stewartii* subsp. *stewartii*	*Zea mays*	Before 2000
CREA-DC 1869	*Pantoea stewartii* subsp. *stewartii*	*Zea mays*	2015
CREA-DC 1870	*Pantoea stewartii* subsp. *stewartii*	*Zea mays*	2015
CREA-DC 1899	*Pantoea stewartii* subsp. *stewartii*	*Zea mays*	2018
CREA-DC 1900	*Pantoea stewartii* subsp. *stewartii*	*Zea mays*	2018
CREA-DC 1923	*Pantoea stewartii* subsp*. indologenes*	*Ananas comosus*	1966
CREA-DC 1924	*Pantoea stewartii* subsp*. indologenes*	*Cyamopsis* sp.	1966

*The strain number refers to the CREA-DC collection.

### 2.4. Phylogenomic analysis

Phylogenetic trees have been performed by using REALPHY software (v1.12) and RaxML (v8.2.12) (Stamatakis, 2014). Anytime, RaxML has been applied, and a maximum likelihood search is adopted for the best tree and evaluated with 1,000 bootstrap iterations by means of the rapid bootstrap algorithm (Stamatakis et al., 2008), employed by RaxML. We chose the general time-reversible model of DNA evolution with the gamma model of rate heterogeneity (GTRGAMMA). All phylogenetic trees have been plotted as cladograms to show the relation between the analyzed strains. The quality of the sequencing process has been checked by the replicates performed for some isolates. The replicates cluster together.

### 2.5. Biochemical and pathogenicity tests

Aesculin hydrolysis and arbutin tests have been performed on pure cultures of every Pss and Psi strain reported in Table 1, *P. agglomerans* CREA-DC 1235, and sweet maize endophyte (i.e., CREA-DC 1875, CREA-DC 1876), according to the European Plant and Protection Organization (2016). *Pseudomonas syringae* pv. *tomato* (CREA-DC 3000) and *Xanthomonas arboricola* pv. *juglandis* (NCPPB 362) have been used as positive controls. The biochemical tests have been repeated at least three times. The pathogenicity test has been performed with 10 plants of sweet maize seedlings F1 (*Z. mays* L. cv. Centurion) of 8–14-day-old (1–2 leaf stages), for each bacterial strain. The plants have been stem inoculated following the European Plant and Protection Organization (2016) and grown in a quarantine glasshouse at 22–28°C. Plants inoculated with all Pss and Psi strains are reported in Table 1, *P. agglomerans* CREA-DC 1235 and sweet maize endophyte (i.e., CREA-DC 1875, CREA-DC 1876). Negative control plants have been inoculated with sterile distilled water. The disease symptoms appeared after 7 days. Plants have been kept for observation for 30 days. Re-isolation from symptomatic tissues has been performed, and Pss-like colonies were identified by real-time PCR TaqMan and real-time PCR SYBR green protocols (Tambong et al., 2008; Pal et al., 2019).

### 2.6. Molecular tests

In order to design primer pairs that would specifically detect Pss, the genomes of approximately 30 Pss and Psi strains found in the NCBI database have been compared to each other to identify a region-specific only in the Pss genome. To increase the analytical specificity and sensitivity, we sought an element that would be unique for the Pss genome and repetitive elements. By using the sequences of three repetitive elements and the primer blast tool, three sets of primers have been designed and preliminarily tested on Psi (CREA-DC 1923, CREA-DC 1924) and Pss (CREA-DC 1775, CREA-DC 1869, CREA-DC 1899) strains. The forward primer ctg3-F -5′- CCG TCA GGG GCT TTG AAT −3′ and reverse primer ctg3-R-5′- GAT GCC AGA CAG AAC ACC GT −3′ have been selected for the molecular test since they can specifically detect Pss and not Psi. The SYBR Green real-time PCR using ctg3 primers has been performed as follows: 4 μl of DNA, 10 μl of 2X Sybr master mix from Applied Biosystems (Thermo Fisher Scientific), 0.8 μl of 10 μM ctg3 forward and reverse primers, and 4.4 μl PCR-grade water in a total reaction volume of 20 μl. Amplification conditions were 95°C for 10 min and 35 cycles of 95°C for 15 s and 65°C for 1 min, followed by a melt curve from 65°C to 95°C in the increment of 0.5°C.

The real-time PCR TaqMan protocol of Tambong et al. (2008) has been performed as reported in the EPPO protocol (2016), employing the Sso Advanced Universal Probes Supermix (Biorad). The primer and probe applied were cps-RT74F 5′ -TGC TGA TTT TAA GTT TTG CTA-3′; cps-177R: 5′ -AAG ATG AGC GAG GTC AGG ATA-3′; probe: cps-133 5′ -TCG GGT TCA CGT CTG TCC AAC T-3′. The SYBR Green real-time of Pal et al. (2019) has been carried out according to instructions received within Test Performance Study (TPS) code: Pstew-1, in the frame of the EU project Valitest (grant agreement N° 773139). The primer pairs applied were cpsAB2313F 5’-AGAAAACGCTGATGCCAGAC-3′ and cpsR- 5’ ACTATCCTGACTCAGGCACT-3′. In brief, the 2X Sybr master mix from Applied Biosystems (Thermo Fisher Scientific, different from the master mix employed within the Pstew-1 TPS) and 4 μl of DNA have been employed for the mix preparation. The thermal profile is 40 cycles at 95°C for 10 min, 95°C for 15 s, and 65°C for 1 min (ramping 1.61C/s), melting curve 65–95°C, 0.05°C/s. In all runs are included one negative amplification control (NAC), which consists of PCR-grade water and one positive amplification control (PAC). All the real-time PCR reactions have been carried out using CFX96 Real-Time System, BioRad. For all the real-time PCR, the standard deviation of the cycle threshold (Ct) values is calculated from the arithmetic mean of three biological replicates and each biological replicate has been amplified in two technical replicates. Conventional PCR has been carried out according to the European Plant and Protection Organization (2016).

### 2.7. Standard curves, analytical sensitivity, and specificity of the ctg3 real-time PCR

Standard curves for ctg3 real-time PCR have been performed by using 10-fold serial dilutions of the Pss genomic DNA (10 ng–10 fg) (sample type 1), and Pss genomic DNA extracted from 10-fold serially diluted bacterial suspension (10^8^–10^1^ CFU/ml) (sample type 2). The Pss strain IPV-BO 2766 has been used for both the above-described standard curves. The analytical sensitivity of ctg3 real-time PCR has been determined by employing DNA extracted from spiked samples (sample type 3). The preparation of the three sample types has been reported in the paragraph named “samples preparation and DNA extraction.” The specificity of ctg3 real-time PCR is determined with the genomic DNA extracted from 1 ml of the bacterial suspension having the concentration of 10^6^ CFU/ml for every bacterial strain reported in Table 1. Repeatability has been verified by testing the samples in duplicate in two independent runs, and PCR amplification efficiency was calculated from the slope of the standard curve using the following formula: E = 100 × [(10^–1/slope^)–1]. The analytical sensitivity, analytical specificity, and repeatability have been performed according to the indication reported by European Plant Protection Organization (2021).

## 3. Results

### 3.1. Sequencing and genome assembly

A robust genomic dataset has been created by sequencing and assembling the genome of different Italian strains of *Pantoea* spp. and *P. stewartii* subspecies of CREA-DC collection. The genomes have been compared based on a phylogenomic approach using the genomes of Pss and Psi strains deposited in the NCBI database. This approach allowed us to properly assign the Italian strains to a specific phylogenetic clade and to generate a new set of primers that can actually discriminate Psi and Pss in a reliable way.

Nine strains including one *P. agglomerans*, two *P. stewartii* subsp. *indologenes*, and six *P. stewartii* subsp. *stewartii* isolated in Italy between 2000 and 2018 have been sequenced with Nanopore MinION (Table 2). Several sequences were larger than 50x coverage of the haploid genome size, which has guaranteed a good quality genome for every tested strain (Table 3). The best genome assembly has been selected for each strain based on the number of contigs generated and on the best largest contig (Table 3). The best assembly was obtained for *P. agglomerans* strain CREA-DC 1235 with only three contigs and the largest of approximately 4 Mb. The best Psi assembly was strain 1923 with only five contigs assembled. All Pss strains have been assembled in more than 10 contigs, with the best being strain 1870 (run 3). Duong et al. (2017) showed that Pss is rich in repetitive elements. Similarly, we found that from 6 to 8% of the genomes of every Pss are constituted of DNA repetitive elements (Supplementary Table S1). Interestingly, Psi does not show almost any repetitive sequences suggesting a different evolutionary trajectory between the two subspecies.

**Table 3:** Genome assemblies’ statistics of sequenced strains.

Genotype	Species name	Sequencing kit	# bases	Genome coverage	# contigs	Largest contig	Total length	GC (%)	N50	L50
CREA-DC 1235^a^	*P*. *agglomerans*	SQK-RBK004	261,671,375	52.33	3	4,017,623	4,843,201	55.20	4,017,623	1
CREA-DC 1775_1	*P*. *stewartii* subsp. *stewartii*	SQK-RBK004	107,807,467	21.56	53	785,459	4,911,472	54.23	167,317	8
CREA-DC 1775_2^a^	*P*. *stewartii* subsp. *stewartii*	SQK-RBK004	156,753,175	31.35	14	4,600,500	5,390,523	53.71	4,600,500	1
CREA-DC 1788^a^	*P*. *stewartii* subsp. *stewartii*	SQK-RBK004	81,487,587	16.30	61	468,927	4,765,329	54.29	137,591	10
CREA-DC 1869_1^a^	*P*. *stewartii* subsp. *stewartii*	SQK-RBK004	135,875,235	27.18	27	1,327,995	5,085,798	54.14	907,223	3
CREA-DC 1869_2	*P*. *stewartii* subsp. *stewartii*	SQK-RBK004	63,460,290	12.69	87	278,060	4,759,659	53.69	98,937	17
CREA-DC 1869_3	*P*. *stewartii* subsp. *stewartii*	SQK-LSK109	173,601,427	34.72	69	549,839	5,361,483	53.73	189,881	9
CREA-DC 1870_1	*P*. *stewartii* subsp. *stewartii*	SQK-RBK004	265,459,724	53.09	100	1,026,647	5,519,910	53.96	562,955	4
CREA-DC 1870_2	*P*. *stewartii* subsp. *stewartii*	SQK-RBK004	251,013,379	50.20	124	1,766,173	5,885,309	53.72	371,824	4
CREA-DC 1870_3^a^	*P*. *stewartii* subsp. *stewartii*	SQK-LSK109	196,282,110	39.26	15	4,115,557	5,422,434	53.64	4,115,557	1
CREA-DC 1899^a^	*P*. *stewartii* subsp. *stewartii*	SQK-RBK004	360,580,303	72.12	18	4,566,110	5,432,163	53.59	4,566,110	1
CREA-DC 1900^a^	*P*. *stewartii* subsp. *stewartii*	SQK-RBK004	171,601,251	34.32	17	2,839,883	5,546,393	53.65	2,839,883	1
CREA-DC 1923^a^	*P*. *stewartii* subsp. *indologenes*	SQK-RBK004	176,167,109	35.23	5	2,405,338	4,847,684	53.62	1,606,128	2
CREA-DC 1924	*P*. *stewartii* subsp. *indologenes*	SQK-RBK004	62,590,469	12.52	1,095	6,758	2,172,097	54.23	2,180	353

^a^Indicate the selected genome assembly.

### 3.2. Phylogenomic analysis reveals multiple Pss introgression in Italy

**Figure 1:**
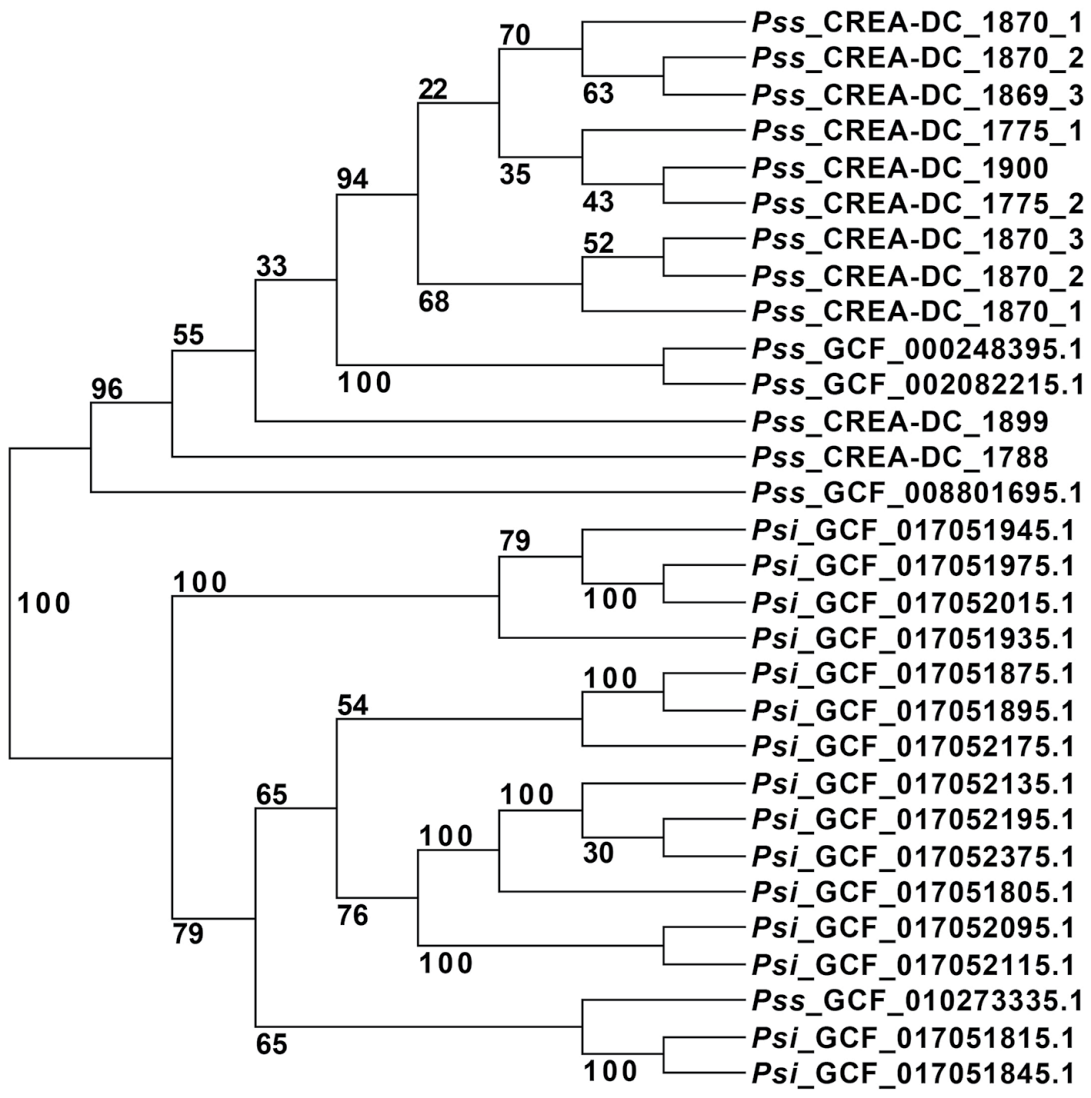
Unrooted phylogenetic tree of all *Pantoea stewartii* present at the NCI database, and the strains sequenced in this study with replicates. The strain GCF_002082215.1 was used as a reference for the alignment in RealPhy.

*Pantoea stewartii* subsp. *stewartii* has been recovered multiple times in Italy during the last few years. The phylogenetic tree showed that multiple runs of sequencing from the same strain are clustered together. To ensure that the differences generated in genomic sequences are not due to the Nanopore error rate, we sequenced some strains three times and generated the phylogenetic tree (Table 3). The results showed that independent sequencing clustered together displayed high reproducibility of the methodology. This aspect suggests that Nanopore reads can be used in the phylogenetic analysis by RealPhy software (Supplementary Figure S1). Therefore, to understand the relationship between the different isolates recovered in Italy during the last few years, all the in-house Pss generated sequences have been compared to the Pss genomes deposited in the NCBI database. One interesting result of the phylogenetic analysis is that the Pss strain MS1, identified by the assembly GCF_010273335.1, clustered between the Psi strains suggesting a misclassification of this strain (Figure 1). To support the misclassification, we look at the number of repeats assembled in the GCF_010273335.1 genome. The data show that the genome of GCF_010273335.1 has only the 0.56% of the genome made of repetitive elements, that is, comparing it to the 7–8% of repetitive elements shown by every other Pss strain (Supplementary Table S2), leads us to hypothesize the genome GCF_010273335.1 belongs to a Psi. Furthermore, the phylogenetic tree clearly shows that the strains isolated in Italy did not cluster together. In between the same clades, other strains isolated in other areas of the world are present (Figure 1). We, here, suggest that Pss has been likely imported into Italy multiple times rather than being representatives of an infection cluster of Pss generated in Italy.

### 3.3. Region selection and primer design

The genome of Pss and Psi strains has been compared to identify regions specific to Pss. Approximately 30 genomes of Pss and Psi have been downloaded from the NCBI database and included in the analysis. As aforementioned, many repetitive elements characterize the Pss genome. Therefore, we chose to design primers on three different repetitive element families. Among these regions, based on their specificity to Pss, we selected the ctg3 pair of primers for further analyzes (Supplementary Figure S2).

### 3.4. Standard curves obtained using ctg3 primers

**Figure 2:**
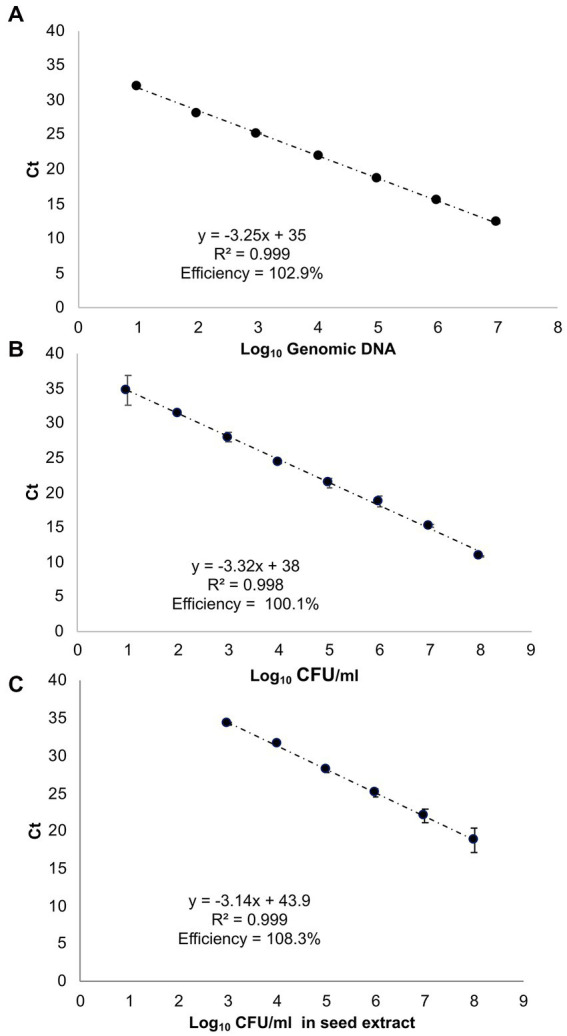
Standard curves of real-time PCR with cycle threshold (Ct) values plotted against **(A)** 10-fold serial dilutions of genomic DNA of Pss strain IPV-BO 2766 (indicated as Log_10_ of fg of genomic DNA); **(B)** 10-fold serial dilutions of the bacterial cell (Log_10_ of CFU/ml); **(C)** the 10-fold serial dilutions of the bacterial cell employed to spike the seed extract (Log_10_ CFU/ml). The Ct value is the mean of three replicates from two independent runs.

The standard curve employing ctg3 real-time PCR and the genomic DNA of Pss (IPV-BO 2766) 10-fold dilution from 10 ng to 10 fg (type 1 of samples) shows a linear correlation (*R*^2^ = 0.999, slope = −3.25) and a PCR efficiency of 102.9% (Figure 2A; Supplementary Figure 3A). The standard curve performed with ctg3 real-time PCR and the genomic DNA extract from 10-fold dilution (from 10^8^ to 10^1^ CFU/ml) of bacterial cells of Pss (IPV-BO 2766; type 2 of samples) showed a linear correlation (*R*^2^ = 0.997, slope = −3.32) and efficacy of 100.1% (Figure 2B; Supplementary Figure S3B). The standard curve performed with ctg3 real-time PCR and the spiked samples (type 3 of samples) are presented in Figure 2C and Supplementary Figure S3C, and the corresponding Ct values are shown in Table 4. The curve shows a linear correlation (*R*^2^ = 0.999, slope = −3.14) and a PCR efficiency of 108.3%.

**Table 4:** Analytical sensitivity of ctg3 compared with Tambong et al., 2008 and Pal et al. (2019) performed on Pss bacterial cell suspension and spiked samples.

Sample CFU/mL	Real-time PCR, mean Ct ± SD *Pss* bacterial cell suspension			Real-time PCR, mean Ct ± SD spiked samples		
	ctg3	Tambong et al. (2008)	Pal et al. (2019)	ctg3	Tambong et al. (2008)	Pal et al. (2019)
10^8^	10.84 ± 0.01	15.58 ± 0.52	12.53 ± 0.2	18.75 ± 0.18	23.19 ± 0.13	21.32 ± 0.24
10^7^	15.17 ± 0.18	19.41 ± 0.34	17.24 ± 0.2	21.98 ± 0.06	26.69 ± 0.88	25.42 ± 0.29
10^6^	18.75 ± 0.82	23.23 ± 0.99	21 ± 0.72	25.07 ± 0.31	29.63 ± 1.43	28.98 ± 1
10^5^	21.38 ± 0.74	25.69 ± 0.08	25.42 ± 0.82	28.07 ± 0.5	32.38 ± 0.91	37.06 ± 0.18
10^4^	24.36 ± 0.12	29.4 ± 0.61	29.01 ± 0.44	31.61 ± 0.97	35.69 ± 1.26	N/A
10^3^	27.9 ± 0.68	34.03 ± 0.44	32.86 ± 0.12	34.34 ± 1.66	N/A	N/A
10^2^	31.41 ± 0.04	36.41 ± 0.83	N/A	N/A	N/A	N/A
10^1^	34.73 ± 2.14	N/A	N/A	N/A	N/A	N/A
0	N/A	N/A	N/A	N/A	N/A	N/A

### 3.5. Sensitivity and specificity of ctg3 SYBR green real-time PCR

The analytical sensitivity of the ctg3 real-time PCR has been determined; the minimum detection level is 10 fg for Pss genomic DNA with sample type 1, with a Ct mean value of 32.01. The analytical sensitivity of ctg3 real-time PCR found using bacterial cell suspension (sample type 2) and spiked samples (type 3) are evaluated and compared with the real-time PCR currently used for the detection of Pss (Tambong et al., 2008; Pal et al., 2019). The analytical sensitivity for sample type 2 by applying the ctg3 real-time PCR was found to be 10^1^ CFU/ml, whereas it was 10^2^ and 10^3^ CFU/ml by applying the real-time PCR protocols of Tambong et al. (2008) and Pal et al. (2019), respectively (Figure 2B; Table 4). The analytical sensitivity of ctg3 real-time PCR with spiked sample type 3 was 10^3^ CFU/ml, whereas it was 10^4^ and 10^5^ CFU/ml following the protocols of Tambong et al. (2008) and Pal et al. (2019), respectively (Figure 2C; Table 4). The analytical specificity (inclusivity and exclusivity) of ctg3 real-time PCR has been evaluated by using the genomic DNA extracted from bacterial suspensions having a concentration of 10^6^ CFU/ml, following the indications as reported in the European Plant and Protection Organization (2021). Every strain (Table 1) has been analyzed to evaluate the ctg3 real-time analytical specificity. The ctg3 real-time PCR specificity assay was 100% for all the strains tested as reported in Table 5. All the Pss strains showed a positive amplification curve with a mean Ct value of 19.8 ± 1.36 and a melting temperature of 77 ± 0.5°C. No amplification (Ct = N/A) was observed for *P. agglomerans*, *P. ananatis*, Psi, all the *Pantoea* spp., the isolates of sweet maize endophytes, and all the non-*Pantoea* isolates (Ct = N/A) (Supplementary Figure S4). The analytical specificity of the ctg3 real-time PCR was evaluated in comparison with Tambong et al. (2008) and Pal et al. (2019) for Pss, Psi, *P. agglomerans*, *P. ananatis*, and *Pantoea* spp. strains and sweet maize endophyte as indicated in Table 5. Ctg3 and the procedure mentioned in Pal et al. (2019) showed 100% analytical specificity (both inclusivity and exclusivity), whereas the procedure mentioned in Tambong et al. (2008) showed an analytical specificity of 56 and 100% for exclusivity and inclusivity, respectively.

**Table 5:** Results of analytical specificity and biochemical and pathogenicity tests.

Species name/CREA-DC code*	Pathogenicity	Biochemical tests		Conventional and real-time PCR methods (methods C is the real-time PCR assay developed in this study)				
		Arbutin	Aesculin hydrolysis	Coplin	Ages	ctg3	Tambong	Pal
*P. stewartii* subsp. s*tewartii*								
CREA-DC 1775	+	−	−	+	+	+	+	+
CREA-DC 1869	+	−	−	+	+	+	+	+
CREA-DC 1870	+	−	−	+	+	+	+	+
CREA-DC 1899	+	−	−	+	+	+	+	+
CREA-DC 1900	+	−	−	+	+	+	+	+
*P. stewartii* subsp. *indologenes*								
CREA-DC 1923	−	+	+	−	NP	−	+	−
CREA-DC 1924	−	+	+	−	NP	−	+	−
*P. agglomerans*								
CREA-DC 1235	−	+	+	−	−	−	−	−
CREA-DC 1939	NP	NP	NP	NP	NP	−	−	−
CREA-DC 2057	NP	NP	NP	NP	NP	−	NP	NP
*P. ananantis*								
CREA-DC 2059	NP	NP	NP	NP	NP	−	−	−
CREA-DC 2060	NP	NP	NP	NP	NP	−	−	−
Sweet maize endophite								
CREA-DC 1868	NP	NP	NP	NP	NP	−	−	−
CREA-DC 1875	−	+	−	−		−	+	−
CREA-DC 1876	−	+	−	NP	NP	−	+	−
CREA-DC 1877				NP	NP	−	−	−
*Pantoea* spp.								
CREA-DC 1925	NP	NP	NP	NP	NP	−	−	−
CREA-DC 1926	NP	NP	NP	NP	NP	−	+	−
CREA-DC 1927	NP	NP	NP	NP	NP	−	−	−
CREA-DC 1928	NP	NP	NP	NP	NP	−	−	−
CREA-DC 1929	NP	NP	NP	NP	NP	−	+	−
CREA-DC 1930	NP	NP	NP	NP	NP	−	+	−
CREA-DC 1931	NP	NP	NP	NP	NP	−	−	−
CREA-DC 1932	NP	NP	NP	NP	NP	−	+	−
*Acidovorax spp.*								
CREA-DC 1852	NP	NP	NP	NP	NP	−	NP	NP
*Brenneria nigrifluens*								
CREA-DC 1830	NP	NP	NP	NP	NP	−	NP	NP
*Brenneria populi*								
CREA-DC 1313	NP	NP	NP	NP	NP	−	NP	
*Clavibacter michiganensis* subsp. *michiganensis*								
CREA-DC 1044	NP	NP	NP	NP	NP	−	NP	NP
*Clavibacter michiganensis* subsp. *Sepedonicus*								
CREA-DC 1041	NP	NP	NP	NP	NP	−	NP	NP
*Erwinia amylovora*								
CREA-DC 1219	NP	NP	NP	NP	NP	−	NP	NP
CREA-DC 1218	NP	NP	NP	NP	NP	−	NP	NP
*Pseudomonas syringae* pv. *tomato*								
CREA-DC 1364	−	+	−	−	−	−	−	−
CREA-DC 1082	NP	NP	NP	NP	NP	−	NP	NP
*Pseudomonas syringae* pv. *actinidiae*								
CREA-DC 1625	NP	NP	NP	NP	NP	−	NP	NP
*Pectobacterium carotovorum*								
CREA-DC 1249	NP	NP	NP	NP	NP	−	NP	NP
*Pectobacterium carotovorum ssp*. *atroseptica*								
CREA-DC 1156	NP	NP	NP	NP	NP	−	NP	NP
*Xanthomonas arboricola* pv. *juglandis*								
CREA-DC 1012	−	−	+	−	−	−	−	−
*Xanthomonas arboricola* pv. *pruni*								
CREA-DC 1151	NP	NP	NP	NP	NP	−	NP	NP
*Xanthomonas campestris* pv. *campestris*								
CREA-DC 1032	NP	NP	NP	NP	NP	−	NP	NP
*Xanthomonas vesicatoria*								
CREA-DC 1855	NP	NP	NP	NP	NP	−	NP	NP
*Xanthomonas gardneri*								
CREA-DC 1856	NP	NP	NP	NP	NP	−	NP	NP
*Xanthomonas euvesicatoria*								
CREA-DC 1857	NP	NP	NP	NP	NP	−	NP	NP
*Xanthomonas perforans*								
CREA-DC 1858	NP	NP	NP	NP	NP	−	NP	NP
*Xanthomonas campestris* pv. *pelargoni*								
CREA-DC 1214	NP	NP	NP	NP	NP	−	NP	NP
*Xylella fastidiosa* subsp. *multiplex*								
CREA-DC 2094	NP	NP	NP	NP	NP	−	NP	NP

Test is not performed (NP). Positive result (+). Negative result (−). Coplin: conventional PCR (Coplin and Majerczak, 2002); Ages: conventional PCR (Euphresco, 2010). ctg3: ctg3 real-time PCR; Tambong: real-time PCR of Tambong et al. (2008); Pal: real-time PCR of Pal et al. (2019).

The analytical specificity of ctg3 real-time PCR is evaluated for all the strains reported in the table. The Pss strains are tested according to the pathogenicity, arbutin, aesculin hydrolysis, cPCR of coplin, and ages as indicated by the European Plant and Protection Organization (2016).

### 3.6. Biochemical and pathogenicity tests

The results of pathogenicity, arbutin and aesculin hydrolysis performed with Pss strains (CREA-DC 1775, CREA-DC 1869, CREA-DC 1870, CREA-DC 1899, and CREA-DC 1900), Psi strain (CREA-DC 1923 and CREA-DC 1924), *P. agglomerans* (CREA-DC 1235), and sweet maize endophytes (CREA-DC 1875 and CREA-DC 1876) are presented in Table 5. All the Pss strains give positive results for the pathogenicity test and negative results for the arbutin and aesculin hydrolysis. This was in accordance with the European Plant and Protection Organization (2016).

## 4. Discussion

Plant pathogenic bacteria represent one of the most important challenges for crop production now and in the very next future. Stewart’s vascular wilt and leaf blight of maize is a disease responsible for serious crop losses (Pepper, 1967; Roper, 2011; CABI, 2019). The causal agent of Pss can be transmitted by infected seed, and in the United States, the pathogen transmission is largely dependent on insect vectors, mainly the flea beetle (*Chaetocnema pulicaria*). Stewart’s wilt is endemic in the mid-Atlantic USA states, the Ohio River Valley, and the southern portion of the Corn Belt. The disease is reported to have declined in prevalence (number of fields in which the pathogen is reported to be present) in the USA due to the use of resistant varieties and the widespread use of neonicotinoid seed treatments.

Despite the low rates of plant-to-seed, seed-to-seedling transmission, and the lack of a known insect vector in the EU, the risk assessment for the entry of Pss through the maize seeds imported by the European Union (EU) from the USA in the order of magnitude of some hundred introductions per year (EFSA Panel on Plant Health, 2019). Pss could become a real threat in Europe (EFSA Panel on Plant Health, 2019). The impacts of Stewarts’ wilt are higher in growing seasons following mild winters, suggesting that “the pathogen should establish and spread in the EU and impacts might worsen in the coming decades due to ongoing climate warming” (EFSA Panel on Plant Health, 2019). The effectiveness of detecting PSs in seed lots depends on the analytical sensitivity of the test and the number of seeds taken for testing (Pataky and Ikin, 2003).

The adoption of reliable detection methods with good performance is crucial to correctly determine the presence of a quarantine pest before its establishment in a pest-free area and/or its spread (EFSA Panel on Plant Health, 2019). The diagnostic protocol reported in the EPPO standard PM 7/60 (2) (European Plant and Protection Organization, 2016) report different molecular tests to detect the presence of Pss in plant and/or seeds. These tests can be used for commercial seed certification and official analysis, but false-positive cross-reactions can occasionally occur when Psi is also present.

*Pantoea stewartii* subsp. *stewartii* was included in the EURL-BAC work program in 2021–2022 with the aim of standardizing test protocols, developing and validating detection, and/or identification tests, providing reference material, and facilitating the disclosure of the procedures for the Pss diagnosis to all the national reference laboratories (NRLs).

The Valitest project funded by the European Union’s Horizon 2020 research and innovation program (GA n◦773,139) organized a test performance study (TPS) with the aim to validate several molecular diagnostic tests for Pss detection in maize seed extracts. The results provided indications of the validated diagnostic methods, highlighting their performance. In particular, it is reported that only the test of Pal et al. (2019) and the conventional PCR of Gehring et al. (2014) can specifically detect Pss. The TPS report (named Pstew-1) indicates that the conventional PCR of Gehring et al. (2014) differentiates Pss and Psi but with low analytical sensitivity. Consequently, it is not reliable for the detection of Pss in maize seeds but can be used as a confirmatory test when Pss concentration is high and/or as an identification test on colonies (Dreo, 2020).

*Pantoea stewartii* subsp. *stewartii* was recovered multiple times in Italy during the last few years. Considering all these aspects, the scarce knowledge about this pathogen at the genome level, and the recent outbreaks of Stewart’s wilt in Italy, in this study, we sequenced the whole genome of Pss strains isolated in Italian territory in 2015 and 2018. Using a comparative genomic approach, we aimed to understand whether the presence of Pss is due to reiterative introductions or to an infection hotspot. The results suggest that Pss is likely imported in Italy multiple times rather than being representative of an infection cluster. This result can suggest that there is a rift in the diagnostic tests that allow the diffusion of Pss by seed trade. This consideration is fundamental to prevent “biological invasion” through the seed market and global trade. Phytosanitary measures undertaken by governments and organizations such as EPPO try to limit the spread of crop pathogens, but in an increasingly connected world for successful management of plant pathogens, it is fundamental to improve the knowledge about pathogen genomes, to apply an early and reliable detection test for a prompt interception of the novel or emerging plant pests.

Our *in silico* analysis identifies a region unique to the Pss genome where ctg3 primers were selected for the development of a real-time PCR able to specifically detect Pss in maize seeds. The ctg3 real-time PCR showed good amplification efficiency and higher analytical sensitivity with respect to the real-time developed by Tambong et al. (2008) and Pal et al. (2019). The difference in the analytical sensitivity of ctg3 real-time PCR among DNA extracts by bacterial cell suspensions (i.e., 10^1^ CFU/ml) and spiked sample (i.e., 10^3^ CFU/ml) was due to the presence of the plant matrix, which may interfere with the amplification reaction. For all the DNA samples analyzed by the real-time PCR by using the ctg3 primers, the calculated efficiencies resulted to be in the acceptable range between 95 and 120%, as well as the resulting R-squared (R2), which were all above 0.95 (Figure 2). Those results suggest the robustness of the real-time PCR method performed with the ctg3 primers. Moreover, the specificity of the ctg3 test was evaluated on Pss strains (inclusivity) and *Pantoea* spp.; the specificity of sweet maize endophyte and non-*Pantoea* isolates (exclusivity) was 100%.

In conclusion, although there are different tests for the detection of Pss with good performance criteria, very few are suitable for the detection of this pathogen in infected maize seeds that do not misidentify Pss when Psi is present. Our test is an additional diagnostic tool, useful for confirmatory and routine testing of Pss in maize seed.

Furthermore, and interestingly, Pss and Psi genome analyzes suggest that further studies are needed to disentangle the role of mobile elements in the host-adaptability of these species since mobile elements could be involved in the host-specialization within these species (Vale et al., 2022).

## Data availability statement

The data presented in the study are deposited in the bioproject repository at https://www.ncbi.nlm.nih.gov/, accession number PRJNA856801.

## Author contributions

VS, SL, LF, and NP contributed to the conception and design of the study. LF, AA, FC, VC, and NP contributed to the methodology of the study. FC performed the statistical analysis. VS, AA, LF, and NP contributed to the investigation of the data. FC, LF, AA, and VS wrote the original draft. SL, MR, and NP contributed to the writing, reviewing, and editing of the manuscript. SL contributed to funding acquisition. All authors contributed to the article and approved the submitted version.

## Funding

This research was funded by MIPAAF, Proteggo 1.4” DISR-05-0001837-04/01/2022 Ministero delle politiche agricole alimentari e forestali–Consiglio per la ricerca in agricoltura e l’analisi dell’economia Agraria.

## Conflict of interest

The authors declare that the research was conducted in the absence of any commercial or financial relationships that could be construed as a potential conflict of interest.

## Publisher’s note

All claims expressed in this article are solely those of the authors and do not necessarily represent those of their affiliated organizations, or those of the publisher, the editors and the reviewers. Any product that may be evaluated in this article, or claim that may be made by its manufacturer, is not guaranteed or endorsed by the publisher.
