# Supplementary material for: Analysis of Italian isolates of Pantoea stewartii subsp. stewartii and development of a real-time PCR-based diagnostic method
Source: Front Microbiol. 2023 Apr 27;14:1129229. doi: 10.3389/fmicb.2023.1129229 (PMC10174441; doi:10.3389/fmicb.2023.1129229)
Supplement: Supplementary file 1 [file Data_Sheet_1.PDF]

Table S1) Report of RepetMasker on all available genomes of Pantoea s

Species
Pantoea agglomerans
Pantoea stewartii subsp. *stewartii*
Pantoea stewartii subsp. *stewartii*
Pantoea stewartii subsp. *stewartii*
Pantoea stewartii subsp. *stewartii*
Pantoea stewartii subsp. *stewartii*
Pantoea stewartii subsp. *stewartii*
Pantoea stewartii subsp. *indologenes*
Pantoea ananatis
Pantoea stewartii subsp. *stewartii*
Pantoea stewartii subsp. *stewartii*
Pantoea stewartii subsp. *stewartii*
Pantoea stewartii subsp. *stewartii*
Pantoea stewartii subsp. *indologenes*
Pantoea stewartii subsp. *indologenes*
Pantoea stewartii subsp. *indologenes*
Pantoea stewartii subsp. *indologenes*
Pantoea stewartii subsp. *indologenes*
Pantoea stewartii subsp. *indologenes*
Pantoea stewartii subsp. *indologenes*
Pantoea stewartii subsp. *indologenes*
Pantoea stewartii subsp. *indologenes*
Pantoea stewartii subsp. *indologenes*
Pantoea stewartii subsp. *indologenes*
Pantoea stewartii subsp. *indologenes*
Pantoea stewartii subsp. *indologenes*
Pantoea stewartii subsp. *indologenes*
Pantoea stewartii subsp. *indologenes*

pp.

Tab
Pa CREA-DC 1235
Pss CREA-DC 1775
Pss CREA-DC 1788
Pss CREA-DC 1869
Pss CREA-DC 1870
Pss CREA-DC 1899
Pss CREA-DC 1900
Psi CREA-DC 1923
Pana_GCF_000233595.1
Pss_GCF_000248395.1
Pss_GCF_002082215.1
Pss_GCF_008801695.1
Pss_GCF_010273335.1
Psi_GCF_017051805.1
Psi_GCF_017051815.1
Psi_GCF_017051875.1
Psi_GCF_017051895.1
Psi_GCF_017051935.1
Psi_GCF_017051945.1
Psi_GCF_017051975.1
Psi_GCF_017052015.1
Psi_GCF_017052095.1
Psi_GCF_017052095.1
Psi_GCF_017052115.1
Psi_GCF_017052135.1
Psi_GCF_017052175.1
Psi_GCF_017052195.1
Psi_GCF_017052375.1

==					
Pa CREA-DC 1235					
sequences	3				
total length	4843201 b	(4843201 bp excl N/X-runs)			
GC level:	55,20%				
bases mas	36062 bp (	0.74 %)			
==					
	number of	length	percentage		
	elements*	occupied	of sequence		
--					
Retroeleme	0	0 bp	0,00%		
	SINEs:	0	0 bp	0,00%	
	Penelope	0	0 bp	0,00%	
	LINEs:	0	0 bp	0,00%	
	CRE/SLAC	0	0 bp	0,00%	
	L2/CR1/Re	0	0 bp	0,00%	
	R1/LOA/Jo	0	0 bp	0,00%	
	R2/R4/NeS	0	0 bp	0,00%	
	RTE/Bov-E	0	0 bp	0,00%	
	L1/CIN4	0	0 bp	0,00%	
	LTR eleme	0	0 bp	0,00%	
	BEL/Pao	0	0 bp	0,00%	
	Ty1/Copia	0	0 bp	0,00%	
	Gypsy/DIR	0	0 bp	0,00%	
	Retroviral	0	0 bp	0,00%	
DNA transp	0	0 bp	0,00%		
	hobo-Activ	0	0 bp	0,00%	
	Tc1-IS630-	0	0 bp	0,00%	
	En-Spm	0	0 bp	0,00%	
	MuDR-IS9(	0	0 bp	0,00%	
	PiggyBac	0	0 bp	0,00%	
	Tourist/Har	0	0 bp	0,00%	
	Other (Mirage		0	0 bp	0,00
	P-element	Transib)			
Rolling-circ	0	0 bp	0,00%		
Unclassifie	80	15046 bp	0,31%		
Total inters	15046 bp	0,31%			
Small RNA	25	10710 bp	0,22%		

Satellites:	0	0 bp	0,00%
Simple rep	224	10128 bp	0,21%
Low compl	4	178 bp	0,00%

==

* most repeats fragmented by insertions or deletions
have been counted as one element

RepeatMas default mode

run with rmblastn version 2.11.0+
The query was compared to classified sequences in ".../1775_20210216_b02.fa-families.fa"
FamDB:

==					
Pss CREA-DC 1775					
sequences	14				
total length	5390523 b	(5390523 bp excl N/X-runs)			
GC level:	53,71%				
bases mas	406944 bp	(7.55 %)			
==					
	number of elements*	length occupied	percentage of sequence		
--					
Retroeleme	0	0 bp	0,00%		
	SINEs:	0	0 bp	0,00%	
	Penelope	0	0 bp	0,00%	
	LINEs:	0	0 bp	0,00%	
	CRE/SLAC	0	0 bp	0,00%	
	L2/CR1/Re	0	0 bp	0,00%	
	R1/LOA/Jo	0	0 bp	0,00%	
	R2/R4/NeS	0	0 bp	0,00%	
	RTE/Bov-B	0	0 bp	0,00%	
	L1/CIN4	0	0 bp	0,00%	
	LTR eleme	0	0 bp	0,00%	
	BEL/Pao	0	0 bp	0,00%	
	Ty1/Copia	0	0 bp	0,00%	
	Gypsy/DIR	0	0 bp	0,00%	
	Retroviral	0	0 bp	0,00%	
DNA trans	78	74719 bp	1,39%		
	hobo-Activ	0	0 bp	0,00%	
	Tc1-IS630-	78	74719 bp	1,39%	
	En-Spm	0	0 bp	0,00%	
	MuDR-IS9	0	0 bp	0,00%	
	PiggyBac	0	0 bp	0,00%	
	Tourist/Har	0	0 bp	0,00%	
	Other (Mirage		0	0 bp	0,00
	P-element	Transib)			
Rolling-circ	0	0 bp	0,00%		
Unclassifie	361	315572 bp	5,85%		
Total inters	390291 bp	7,24%			
Small RNA	25	10604 bp	0,20%		

Satellites:	0	0 bp	0,00%
Simple rep	126	5678 bp	0,11%
Low compl	9	371 bp	0,01%

==

* most repeats fragmented by insertions or deletions
have been counted as one element

RepeatMas default mode

run with rmblastn version 2.11.0+
The query was compared to classified sequences in ".../1775_20210216_b02.fa-families.fa"
FamDB:

==					
Pss CREA-DC 1788					
sequences	61				
total length	4765329 b|	(4765329 bp excl N/X-runs)			
GC level:	54,29%				
bases mas	311069 bp	(6.53 %)			
==					
	number of elements*	length occupied	percentage of sequence		
--					
Retroeleme	0	0 bp	0,00%		
	SINEs:	0	0 bp	0,00%	
	Penelope	0	0 bp	0,00%	
	LINEs:	0	0 bp	0,00%	
	CRE/SLAC	0	0 bp	0,00%	
	L2/CR1/Re	0	0 bp	0,00%	
	R1/LOA/Jo	0	0 bp	0,00%	
	R2/R4/NeS	0	0 bp	0,00%	
	RTE/Bov-B	0	0 bp	0,00%	
	L1/CIN4	0	0 bp	0,00%	
	LTR eleme	0	0 bp	0,00%	
	BEL/Pao	0	0 bp	0,00%	
	Ty1/Copia	0	0 bp	0,00%	
	Gypsy/DIR	0	0 bp	0,00%	
	Retroviral	0	0 bp	0,00%	
DNA transp	50	51263 bp	1,08%		
	hobo-Activ	0	0 bp	0,00%	
	Tc1-IS630-	50	51263 bp	1,08%	
	En-Spm	0	0 bp	0,00%	
	MuDR-IS90	0	0 bp	0,00%	
	PiggyBac	0	0 bp	0,00%	
	Tourist/Har	0	0 bp	0,00%	
	Other (Mirage		0	0 bp	0,00
	P-element	Transib)			
Rolling-circ	0	0 bp	0,00%		
Unclassifie	271	243912 bp	5,12%		
Total inters	295175 bp	6,19%			
Small RNA	24	10610 bp	0,22%		

Satellites:	0	0 bp	0,00%
Simple rep	111	4981 bp	0,10%
Low compl	7	303 bp	0,01%

==

* most repeats fragmented by insertions or deletions
have been counted as one element

RepeatMas default mode

run with rmblastn version 2.11.0+
The query was compared to classified sequences in ".../1775_20210216_b02.fa-families.fa"
FamDB:

==

Pss CREA-DC 1869					
sequences	69				
total length	5361483 b	(5361483 bp excl N/X-runs)			
GC level:	53,73%				
bases mas	400450 bp	(7.47 %)			

==

	number of elements*	length occupied	percentage of sequence		

Retroeleme	0	0 bp	0,00%		
	SINEs:	0	0 bp	0,00%	
	Penelope	0	0 bp	0,00%	
	LINEs:	0	0 bp	0,00%	
	CRE/SLAC	0	0 bp	0,00%	
	L2/CR1/Re	0	0 bp	0,00%	
	R1/LOA/Jo	0	0 bp	0,00%	
	R2/R4/NeS	0	0 bp	0,00%	
	RTE/Bov-B	0	0 bp	0,00%	
	L1/CIN4	0	0 bp	0,00%	
	LTR eleme	0	0 bp	0,00%	
	BEL/Pao	0	0 bp	0,00%	
	Ty1/Copia	0	0 bp	0,00%	
	Gypsy/DIR	0	0 bp	0,00%	
	Retroviral	0	0 bp	0,00%	
DNA trans	72	70197 bp	1,31%		
	hobo-Activ	0	0 bp	0,00%	
	Tc1-IS630-	72	70197 bp	1,31%	
	En-Spm	0	0 bp	0,00%	
	MuDR-IS9	0	0 bp	0,00%	
	PiggyBac	0	0 bp	0,00%	
	Tourist/Har	0	0 bp	0,00%	
	Other (Mirage		0	0 bp	0,00
	P-element	Transib)			
Rolling-circ	0	0 bp	0,00%		
Unclassifie	361	314056 bp	5,86%		
Total inters	384253 bp	7,17%			
Small RNA	20	10194 bp	0,19%		

Satellites:	0	0 bp	0,00%
Simple rep	127	5647 bp	0,11%
Low compl	8	356 bp	0,01%

==

* most repeats fragmented by insertions or deletions
have been counted as one element

RepeatMas default mode

run with rmblastn version 2.11.0+
The query was compared to classified sequences in ".../1775_20210216_b02.fa-families.fa"
FamDB:

==					
Pss CREA-DC 1870					
sequences	15				
total length	5422434 b|	(5422434 bp excl N/X-runs)			
GC level:	53,64%				
bases mas	417641 bp	(7.70 %)			
==					
	number of elements*	length occupied	percentage of sequence		
--					
Retroeleme	0	0 bp	0,00%		
	SINEs:	0	0 bp	0,00%	
	Penelope	0	0 bp	0,00%	
	LINEs:	0	0 bp	0,00%	
	CRE/SLAC	0	0 bp	0,00%	
	L2/CR1/Re	0	0 bp	0,00%	
	R1/LOA/Jo	0	0 bp	0,00%	
	R2/R4/NeS	0	0 bp	0,00%	
	RTE/Bov-B	0	0 bp	0,00%	
	L1/CIN4	0	0 bp	0,00%	
	LTR eleme	0	0 bp	0,00%	
	BEL/Pao	0	0 bp	0,00%	
	Ty1/Copia	0	0 bp	0,00%	
	Gypsy/DIR	0	0 bp	0,00%	
	Retroviral	0	0 bp	0,00%	
DNA trans|	78	75859 bp	1,40%		
	hobo-Activ	0	0 bp	0,00%	
	Tc1-IS630-	78	75859 bp	1,40%	
	En-Spm	0	0 bp	0,00%	
	MuDR-IS9(	0	0 bp	0,00%	
	PiggyBac	0	0 bp	0,00%	
	Tourist/Har	0	0 bp	0,00%	
	Other (Mirage		0	0 bp	0,00
	P-element	Transib)			
Rolling-circ	0	0 bp	0,00%		
Unclassifie	373	324830 bp	5,99%		
Total inters	400689 bp	7,39%			
Small RNA	25	10605 bp	0,20%		

Satellites:	0	0 bp	0,00%
Simple rep	136	6107 bp	0,11%
Low compl	5	240 bp	0,00%

==

* most repeats fragmented by insertions or deletions
have been counted as one element

RepeatMas default mode

run with rmblastn version 2.11.0+
The query was compared to classified sequences in ".../1775_20210216_b02.fa-families.fa"
FamDB:

==					
Pss CREA-DC 1899					
sequences	18				
total length	5432163 b	(5432163 bp excl N/X-runs)			
GC level:	53,59%				
bases mas	424262 bp	(7.81 %)			
==					
	number of elements*	length occupied	percentage of sequence		
--					
Retroeleme	0	0 bp	0,00%		
	SINEs:	0	0 bp	0,00%	
	Penelope	0	0 bp	0,00%	
	LINEs:	0	0 bp	0,00%	
	CRE/SLAC	0	0 bp	0,00%	
	L2/CR1/Re	0	0 bp	0,00%	
	R1/LOA/Jo	0	0 bp	0,00%	
	R2/R4/NeS	0	0 bp	0,00%	
	RTE/Bov-B	0	0 bp	0,00%	
	L1/CIN4	0	0 bp	0,00%	
	LTR eleme	0	0 bp	0,00%	
	BEL/Pao	0	0 bp	0,00%	
	Ty1/Copia	0	0 bp	0,00%	
	Gypsy/DIR	0	0 bp	0,00%	
	Retroviral	0	0 bp	0,00%	
DNA transp	80	77782 bp	1,43%		
	hobo-Activ	0	0 bp	0,00%	
	Tc1-IS630-	80	77782 bp	1,43%	
	En-Spm	0	0 bp	0,00%	
	MuDR-IS9(	0	0 bp	0,00%	
	PiggyBac	0	0 bp	0,00%	
	Tourist/Har	0	0 bp	0,00%	
	Other (Mirage P-element	Transib)	0	0 bp	0,00
Rolling-circ	0	0 bp	0,00%		
Unclassifie	374	330028 bp	6,08%		
Total inters	407810 bp	7,51%			
Small RNA	25	10608 bp	0,20%		

Satellites:	0	0 bp	0,00%
Simple rep	130	5567 bp	0,10%
Low compl	6	277 bp	0,01%

==

* most repeats fragmented by insertions or deletions
have been counted as one element

RepeatMas default mode

run with rmblastn version 2.11.0+
The query was compared to classified sequences in ".../1775_20210216_b02.fa-families.fa"
FamDB:

==
Pss CREA-DC 1900
sequences 17
total length 5546393 bp (5546393 bp excl N/X-runs)
GC level: 53,65%
bases mas 415998 bp (7.50 %)
==

		number of elements*	length occupied	percentage of sequence
Retroeleme		0	0 bp	0,00%
	SINEs:	0	0 bp	0,00%
	Penelope	0	0 bp	0,00%
	LINEs:	0	0 bp	0,00%
	CRE/SLAC	0	0 bp	0,00%
	L2/CR1/Re	0	0 bp	0,00%
	R1/LOA/Jo	0	0 bp	0,00%
	R2/R4/NeS	0	0 bp	0,00%
	RTE/Bov-B	0	0 bp	0,00%
	L1/CIN4	0	0 bp	0,00%
	LTR eleme	0	0 bp	0,00%
	BEL/Pao	0	0 bp	0,00%
	Ty1/Copia	0	0 bp	0,00%
	Gypsy/DIR	0	0 bp	0,00%
	Retroviral	0	0 bp	0,00%
DNA transp		77	74724 bp	1,35%
	hobo-Activ	0	0 bp	0,00%
	Tc1-IS630-	77	74724 bp	1,35%
	En-Spm	0	0 bp	0,00%
	MuDR-IS90	0	0 bp	0,00%
	PiggyBac	0	0 bp	0,00%
	Tourist/Har	0	0 bp	0,00%
	Other (Mirage P-element Transib)	0	0 bp	0,00
Rolling-circ		0	0 bp	0,00%
Unclassifie		370	324592 bp	5,85%
Total inters		399316 bp	7,20%	
Small RNA		25	10614 bp	0,19%

Satellites:	0	0 bp	0,00%
Simple rep	126	5631 bp	0,10%
Low compl	10	437 bp	0,01%

==

* most repeats fragmented by insertions or deletions
have been counted as one element

RepeatMas default mode

run with rmblastn version 2.11.0+
The query was compared to classified sequences in ".../1775_20210216_b02.fa-families.fa"
FamDB:

==
Psi CREA-DC 1923
sequences 5
total length 4847684 b (4847684 bp excl N/X-runs)
GC level: 53,62%
bases mas 39143 bp (0.81 %)
==

	number of elements*	length occupied	percentage of sequence

Retroeleme 0 0 bp 0,00%
SINEs: 0 0 bp 0,00%
Penelope 0 0 bp 0,00%
LINEs: 0 0 bp 0,00%
CRE/SLAC 0 0 bp 0,00%
L2/CR1/Re 0 0 bp 0,00%
R1/LOA/Jo 0 0 bp 0,00%
R2/R4/NeS 0 0 bp 0,00%
RTE/Bov-B 0 0 bp 0,00%
L1/CIN4 0 0 bp 0,00%
LTR eleme 0 0 bp 0,00%
BEL/Pao 0 0 bp 0,00%
Ty1/Copia 0 0 bp 0,00%
Gypsy/DIR 0 0 bp 0,00%
Retroviral 0 0 bp 0,00%

DNA trans 0 0 bp 0,00%
hobo-Activ 0 0 bp 0,00%
Tc1-IS630- 0 0 bp 0,00%
En-Spm 0 0 bp 0,00%
MuDR-IS9 0 0 bp 0,00%
PiggyBac 0 0 bp 0,00%
Tourist/Har 0 0 bp 0,00%
Other (Mirage 0 0 bp 0,00
P-element Transib)

Rolling-circ 0 0 bp 0,00%

Unclassifie 85 23371 bp 0,48%

Total inters 23371 bp 0,48%

Small RNA 26 10670 bp 0,22%

Satellites:	0	0 bp	0,00%
Simple rep	117	5004 bp	0,10%
Low compl	3	98 bp	0,00%

==

* most repeats fragmented by insertions or deletions
have been counted as one element

RepeatMas default mode

run with rmblastn version 2.11.0+
The query was compared to classified sequences in ".../1775_20210216_b02.fa-families.fa"
FamDB:

==
Pana_GCF_000233595.1

sequences	2		
total length	4867131 b|	(4867131 bp excl N/X-runs)	
GC level:	53,58%		
bases mas	37671 bp (	0.77 %)	

==

	number of elements*	length occupied	percentage of sequence		
Retroeleme	0	0 bp	0,00%		
	SINEs:	0	0 bp	0,00%	
	Penelope	0	0 bp	0,00%	
	LINEs:	0	0 bp	0,00%	
	CRE/SLAC	0	0 bp	0,00%	
	L2/CR1/Re	0	0 bp	0,00%	
	R1/LOA/Jo	0	0 bp	0,00%	
	R2/R4/NeS	0	0 bp	0,00%	
	RTE/Bov-B	0	0 bp	0,00%	
	L1/CIN4	0	0 bp	0,00%	
	LTR eleme	0	0 bp	0,00%	
	BEL/Pao	0	0 bp	0,00%	
	Ty1/Copia	0	0 bp	0,00%	
	Gypsy/DIR	0	0 bp	0,00%	
	Retroviral	0	0 bp	0,00%	
DNA transp	0	0 bp	0,00%		
	hobo-Activ	0	0 bp	0,00%	
	Tc1-IS630-	0	0 bp	0,00%	
	En-Spm	0	0 bp	0,00%	
	MuDR-IS9(	0	0 bp	0,00%	
	PiggyBac	0	0 bp	0,00%	
	Tourist/Har	0	0 bp	0,00%	
	Other (Mirage P-element Transib)		0	0 bp	0,00
Rolling-circ	0	0 bp	0,00%		
Unclassifie	71	18820 bp	0,39%		
Total inters	18820 bp	0,39%			
Small RNA	26	12302 bp	0,25%		

Satellites:	0	0 bp	0,00%
Simple rep	130	6108 bp	0,13%
Low compl	10	441 bp	0,01%

==

* most repeats fragmented by insertions or deletions
have been counted as one element

RepeatMas default mode

run with rmblastn version 2.11.0+
The query was compared to classified sequences in ".../1775_20210216_b02.fa-families.fa"
FamDB:

==
Pss_GCF_000248395.1
sequences 65
total length 5233214 b| (5233214 bp excl N/X-runs)
GC level: 53,84%
bases mas 409795 bp (7.83 %)
==

	number of elements*	length occupied	percentage of sequence		
Retroeleme	0	0 bp	0,00%		
	SINEs:	0	0 bp	0,00%	
	Penelope	0	0 bp	0,00%	
	LINEs:	0	0 bp	0,00%	
	CRE/SLAC	0	0 bp	0,00%	
	L2/CR1/Re	0	0 bp	0,00%	
	R1/LOA/Jo	0	0 bp	0,00%	
	R2/R4/NeS	0	0 bp	0,00%	
	RTE/Bov-B	0	0 bp	0,00%	
	L1/CIN4	0	0 bp	0,00%	
	LTR eleme	0	0 bp	0,00%	
	BEL/Pao	0	0 bp	0,00%	
	Ty1/Copia	0	0 bp	0,00%	
	Gypsy/DIR	0	0 bp	0,00%	
	Retroviral	0	0 bp	0,00%	
DNA transp	77	74378 bp	1,42%		
	hobo-Activ	0	0 bp	0,00%	
	Tc1-IS630-	77	74378 bp	1,42%	
	En-Spm	0	0 bp	0,00%	
	MuDR-IS9(	0	0 bp	0,00%	
	PiggyBac	0	0 bp	0,00%	
	Tourist/Har	0	0 bp	0,00%	
	Other (Mirage P-element Transib)		0	0 bp	0,00
Rolling-circ	0	0 bp	0,00%		
Unclassifie	388	324097 bp	6,19%		
Total inters	398475 bp	7,61%			
Small RNA	25	5012 bp	0,10%		

Satellites:	0	0 bp	0,00%
Simple rep	137	6012 bp	0,11%
Low compl	7	296 bp	0,01%

==

* most repeats fragmented by insertions or deletions
have been counted as one element

RepeatMas default mode

run with rmblastn version 2.11.0+
The query was compared to classified sequences in ".../1775_20210216_b02.fa-families.fa"
FamDB:

==					
Pss_GCF_002082215.1					
sequences	12				
total length	5314092 b|	(5314092 bp excl N/X-runs)			
GC level:	53,77%				
bases mas	414326 bp	(7.80 %)			
==					
	number of elements*	length occupied	percentage of sequence		
--					
Retroeleme	0	0 bp	0,00%		
	SINEs:	0	0 bp	0,00%	
	Penelope	0	0 bp	0,00%	
	LINEs:	0	0 bp	0,00%	
	CRE/SLAC	0	0 bp	0,00%	
	L2/CR1/Re	0	0 bp	0,00%	
	R1/LOA/Jo	0	0 bp	0,00%	
	R2/R4/NeS	0	0 bp	0,00%	
	RTE/Bov-B	0	0 bp	0,00%	
	L1/CIN4	0	0 bp	0,00%	
	LTR eleme	0	0 bp	0,00%	
	BEL/Pao	0	0 bp	0,00%	
	Ty1/Copia	0	0 bp	0,00%	
	Gypsy/DIR	0	0 bp	0,00%	
	Retroviral	0	0 bp	0,00%	
DNA transp	78	75148 bp	1,41%		
	hobo-Activ	0	0 bp	0,00%	
	Tc1-IS630-	78	75148 bp	1,41%	
	En-Spm	0	0 bp	0,00%	
	MuDR-IS9(	0	0 bp	0,00%	
	PiggyBac	0	0 bp	0,00%	
	Tourist/Har	0	0 bp	0,00%	
	Other (Mirage		0	0 bp	0,00
	P-element	Transib)			
Rolling-circ	0	0 bp	0,00%		
Unclassifie	374	322665 bp	6,07%		
Total inters	397813 bp	7,49%			
Small RNA	27	10734 bp	0,20%		

Satellites:	0	0 bp	0,00%
Simple rep	124	5442 bp	0,10%
Low compl	8	337 bp	0,01%

==

* most repeats fragmented by insertions or deletions
have been counted as one element

RepeatMas default mode

run with rmblastn version 2.11.0+
The query was compared to classified sequences in ".../1775_20210216_b02.fa-families.fa"
FamDB:

==

Pss_GCF_008801695.1					
sequences	352				
total length	4916637 b	(4916637 bp excl N/X-runs)			
GC level:	53,57%				
bases mas	130679 bp	(2.66 %)			

==

	number of elements*	length occupied	percentage of sequence		

Retroeleme	0	0 bp	0,00%		
	SINEs:	0	0 bp	0,00%	
	Penelope	0	0 bp	0,00%	
	LINEs:	0	0 bp	0,00%	
	CRE/SLAC	0	0 bp	0,00%	
	L2/CR1/Re	0	0 bp	0,00%	
	R1/LOA/Jo	0	0 bp	0,00%	
	R2/R4/NeS	0	0 bp	0,00%	
	RTE/Bov-B	0	0 bp	0,00%	
	L1/CIN4	0	0 bp	0,00%	
	LTR eleme	0	0 bp	0,00%	
	BEL/Pao	0	0 bp	0,00%	
	Ty1/Copia	0	0 bp	0,00%	
	Gypsy/DIR	0	0 bp	0,00%	
	Retroviral	0	0 bp	0,00%	
DNA transp	139	28124 bp	0,57%		
	hobo-Activ	0	0 bp	0,00%	
	Tc1-IS630-	139	28124 bp	0,57%	
	En-Spm	0	0 bp	0,00%	
	MuDR-IS9(	0	0 bp	0,00%	
	PiggyBac	0	0 bp	0,00%	
	Tourist/Har	0	0 bp	0,00%	
	Other (Mirage		0	0 bp	0,00
	P-element	Transib)			
Rolling-circ	0	0 bp	0,00%		
Unclassifie	496	93150 bp	1,89%		
Total inters	121274 bp	2,47%			
Small RNA	24	3559 bp	0,07%		

Satellites:	0	0 bp	0,00%
Simple rep	124	5543 bp	0,11%
Low compl	7	303 bp	0,01%

==

* most repeats fragmented by insertions or deletions
have been counted as one element

RepeatMas default mode

run with rmblastn version 2.11.0+
The query was compared to classified sequences in ".../1775_20210216_b02.fa-families.fa"
FamDB:

==
Pss_GCF_010273335.1
sequences 59
total length 4780534 b| (4780534 bp excl N/X-runs)
GC level: 53,66%
bases mas 32888 bp (0.69 %)
==

	number of elements*	length occupied	percentage of sequence	
Retroeleme	0	0 bp	0,00%	
SINEs:	0	0 bp	0,00%	
Penelope	0	0 bp	0,00%	
LINEs:	0	0 bp	0,00%	
CRE/SLAC	0	0 bp	0,00%	
L2/CR1/Re	0	0 bp	0,00%	
R1/LOA/Jo	0	0 bp	0,00%	
R2/R4/NeS	0	0 bp	0,00%	
RTE/Bov-B	0	0 bp	0,00%	
L1/CIN4	0	0 bp	0,00%	
LTR eleme	0	0 bp	0,00%	
BEL/Pao	0	0 bp	0,00%	
Ty1/Copia	0	0 bp	0,00%	
Gypsy/DIR	0	0 bp	0,00%	
Retroviral	0	0 bp	0,00%	
DNA trans|	0	0 bp	0,00%	
hobo-Activ	0	0 bp	0,00%	
Tc1-IS630-	0	0 bp	0,00%	
En-Spm	0	0 bp	0,00%	
MuDR-IS9(	0	0 bp	0,00%	
PiggyBac	0	0 bp	0,00%	
Tourist/Har	0	0 bp	0,00%	
Other (Mirage P-element Transib)		0	0 bp	0,00
Rolling-circ	0	0 bp	0,00%	
Unclassifie	84	24454 bp	0,51%	
Total inters	24454 bp	0,51%		
Small RNA	21	3013 bp	0,06%	

Satellites:	0	0 bp	0,00%
Simple rep	118	5031 bp	0,11%
Low compl	9	390 bp	0,01%

==

* most repeats fragmented by insertions or deletions
have been counted as one element

RepeatMas default mode

run with rmblastn version 2.11.0+
The query was compared to classified sequences in ".../1775_20210216_b02.fa-families.fa"
FamDB:

==
Psi_GCF_017051805.1
sequences 26
total length 4687505 b| (4687505 bp excl N/X-runs)
GC level: 53,70%
bases mas 33082 bp (0.71 %)
==

	number of elements*	length occupied	percentage of sequence	
Retroeleme	0	0 bp	0,00%	
SINEs:	0	0 bp	0,00%	
Penelope	0	0 bp	0,00%	
LINEs:	0	0 bp	0,00%	
CRE/SLAC	0	0 bp	0,00%	
L2/CR1/Re	0	0 bp	0,00%	
R1/LOA/Jo	0	0 bp	0,00%	
R2/R4/NeS	0	0 bp	0,00%	
RTE/Bov-B	0	0 bp	0,00%	
L1/CIN4	0	0 bp	0,00%	
LTR eleme	0	0 bp	0,00%	
BEL/Pao	0	0 bp	0,00%	
Ty1/Copia	0	0 bp	0,00%	
Gypsy/DIR	0	0 bp	0,00%	
Retroviral	0	0 bp	0,00%	
DNA transp	1	156 bp	0,00%	
hobo-Activ	0	0 bp	0,00%	
Tc1-IS630-	1	156 bp	0,00%	
En-Spm	0	0 bp	0,00%	
MuDR-IS9(	0	0 bp	0,00%	
PiggyBac	0	0 bp	0,00%	
Tourist/Har	0	0 bp	0,00%	
Other (Mirage P-element Transib)	0	0 bp	0,00	
Rolling-circ	0	0 bp	0,00%	
Unclassifie	87	23806 bp	0,51%	
Total inters	23962 bp	0,51%		
Small RNA	22	2761 bp	0,06%	

Satellites:	0	0 bp	0,00%
Simple rep	130	5700 bp	0,12%
Low compl	13	659 bp	0,01%

==

* most repeats fragmented by insertions or deletions
have been counted as one element

RepeatMas default mode

run with rmblastn version 2.11.0+
The query was compared to classified sequences in ".../1775_20210216_b02.fa-families.fa"
FamDB:

==
Psi_GCF_017051815.1
sequences 28
total length 4857718 bp (4857718 bp excl N/X-runs)
GC level: 53,43%
bases mas 30748 bp (0.63 %)
==

	number of elements*	length occupied	percentage of sequence	
Retroeleme	0	0 bp	0,00%	
SINEs:	0	0 bp	0,00%	
Penelope	0	0 bp	0,00%	
LINEs:	0	0 bp	0,00%	
CRE/SLAC	0	0 bp	0,00%	
L2/CR1/Re	0	0 bp	0,00%	
R1/LOA/Jo	0	0 bp	0,00%	
R2/R4/NeS	0	0 bp	0,00%	
RTE/Bov-B	0	0 bp	0,00%	
L1/CIN4	0	0 bp	0,00%	
LTR eleme	0	0 bp	0,00%	
BEL/Pao	0	0 bp	0,00%	
Ty1/Copia	0	0 bp	0,00%	
Gypsy/DIR	0	0 bp	0,00%	
Retroviral	0	0 bp	0,00%	
DNA transp	1	174 bp	0,00%	
hobo-Activ	0	0 bp	0,00%	
Tc1-IS630-	1	174 bp	0,00%	
En-Spm	0	0 bp	0,00%	
MuDR-IS90	0	0 bp	0,00%	
PiggyBac	0	0 bp	0,00%	
Tourist/Har	0	0 bp	0,00%	
Other (Mirage P-element Transib)	0	0 bp		0,00
Rolling-circ	0	0 bp	0,00%	
Unclassifie	82	21442 bp	0,44%	
Total inters	21616 bp	0,44%		
Small RNA	20	3168 bp	0,07%	

Satellites:	0	0 bp	0,00%
Simple rep	130	5514 bp	0,11%
Low compl	10	450 bp	0,01%

==

* most repeats fragmented by insertions or deletions
have been counted as one element

RepeatMas default mode

run with rmblastn version 2.11.0+
The query was compared to classified sequences in ".../1775_20210216_b02.fa-families.fa"
FamDB:

==
Psi_GCF_017051845.1
sequences 30
total length 4858306 bp (4858306 bp excl N/X-runs)
GC level: 53,43%
bases mas 30896 bp (0.64 %)
==

		number of elements*	length occupied	percentage of sequence
Retroeleme		0	0 bp	0,00%
	SINEs:	0	0 bp	0,00%
	Penelope	0	0 bp	0,00%
	LINEs:	0	0 bp	0,00%
	CRE/SLAC	0	0 bp	0,00%
	L2/CR1/Re	0	0 bp	0,00%
	R1/LOA/Jo	0	0 bp	0,00%
	R2/R4/NeS	0	0 bp	0,00%
	RTE/Bov-E	0	0 bp	0,00%
	L1/CIN4	0	0 bp	0,00%
	LTR eleme	0	0 bp	0,00%
	BEL/Pao	0	0 bp	0,00%
	Ty1/Copia	0	0 bp	0,00%
	Gypsy/DIR	0	0 bp	0,00%
	Retroviral	0	0 bp	0,00%
DNA transp		1	174 bp	0,00%
	hobo-Activ	0	0 bp	0,00%
	Tc1-IS630-	1	174 bp	0,00%
	En-Spm	0	0 bp	0,00%
	MuDR-IS9(	0	0 bp	0,00%
	PiggyBac	0	0 bp	0,00%
	Tourist/Har	0	0 bp	0,00%
	Other (Mirage P-element Transib)	0	0 bp	0,00
Rolling-circ		0	0 bp	0,00%
Unclassifie		82	21442 bp	0,44%
Total inters		21616 bp	0,44%	
Small RNA		22	3316 bp	0,07%

Satellites:	0	0 bp	0,00%
Simple rep	130	5514 bp	0,11%
Low compl	10	450 bp	0,01%

==

* most repeats fragmented by insertions or deletions
have been counted as one element

RepeatMas default mode

run with rmblastn version 2.11.0+
The query was compared to classified sequences in ".../1775_20210216_b02.fa-families.fa"
FamDB:

==					
Psi_GCF_017051875.1					
sequences	36				
total length	4762219 b	(4762219 bp excl N/X-runs)			
GC level:	53,60%				
bases mas	34956 bp (	0.73 %)			
==					
	number of elements*	length occupied	percentage of sequence		
--					
Retroeleme	0	0 bp	0,00%		
	SINEs:	0	0 bp	0,00%	
	Penelope	0	0 bp	0,00%	
	LINEs:	0	0 bp	0,00%	
	CRE/SLAC	0	0 bp	0,00%	
	L2/CR1/Re	0	0 bp	0,00%	
	R1/LOA/Jo	0	0 bp	0,00%	
	R2/R4/NeS	0	0 bp	0,00%	
	RTE/Bov-E	0	0 bp	0,00%	
	L1/CIN4	0	0 bp	0,00%	
	LTR eleme	0	0 bp	0,00%	
	BEL/Pao	0	0 bp	0,00%	
	Ty1/Copia	0	0 bp	0,00%	
	Gypsy/DIR	0	0 bp	0,00%	
	Retroviral	0	0 bp	0,00%	
DNA transp	0	0 bp	0,00%		
	hobo-Activ	0	0 bp	0,00%	
	Tc1-IS630-	0	0 bp	0,00%	
	En-Spm	0	0 bp	0,00%	
	MuDR-IS9(	0	0 bp	0,00%	
	PiggyBac	0	0 bp	0,00%	
	Tourist/Har	0	0 bp	0,00%	
	Other (Mirage		0	0 bp	0,00
	P-element	Transib)			
Rolling-circ	0	0 bp	0,00%		
Unclassifie	85	26401 bp	0,55%		
Total inters	26401 bp	0,55%			
Small RNA	19	3002 bp	0,06%		

Satellites:	0	0 bp	0,00%
Simple rep	119	5195 bp	0,11%
Low compl	8	358 bp	0,01%

==

* most repeats fragmented by insertions or deletions
have been counted as one element

RepeatMas default mode

run with rmblastn version 2.11.0+
The query was compared to classified sequences in ".../1775_20210216_b02.fa-families.fa"
FamDB:

==

Psi_GCF_017051895.1					
sequences	32				
total length	4762030 b	(4762030 bp excl N/X-runs)			
GC level:	53,60%				
bases mas	34830 bp (	0.73 %)			

==

	number of elements*	length occupied	percentage of sequence		

Retroeleme	0	0 bp	0,00%		
	SINEs:	0	0 bp	0,00%	
	Penelope	0	0 bp	0,00%	
	LINEs:	0	0 bp	0,00%	
	CRE/SLAC	0	0 bp	0,00%	
	L2/CR1/Re	0	0 bp	0,00%	
	R1/LOA/Jo	0	0 bp	0,00%	
	R2/R4/NeS	0	0 bp	0,00%	
	RTE/Bov-B	0	0 bp	0,00%	
	L1/CIN4	0	0 bp	0,00%	
	LTR eleme	0	0 bp	0,00%	
	BEL/Pao	0	0 bp	0,00%	
	Ty1/Copia	0	0 bp	0,00%	
	Gypsy/DIR	0	0 bp	0,00%	
	Retroviral	0	0 bp	0,00%	
DNA trans	0	0 bp	0,00%		
	hobo-Activ	0	0 bp	0,00%	
	Tc1-IS630-	0	0 bp	0,00%	
	En-Spm	0	0 bp	0,00%	
	MuDR-IS9	0	0 bp	0,00%	
	PiggyBac	0	0 bp	0,00%	
	Tourist/Har	0	0 bp	0,00%	
	Other (Mirage		0	0 bp	0,00
	P-element	Transib)			
Rolling-circ	0	0 bp	0,00%		
Unclassifie	86	26508 bp	0,56%		
Total inters	26508 bp	0,56%			
Small RNA	22	2783 bp	0,06%		

Satellites:	0	0 bp	0,00%
Simple rep	119	5181 bp	0,11%
Low compl	8	358 bp	0,01%

==

* most repeats fragmented by insertions or deletions
have been counted as one element

RepeatMas default mode

run with rmblastn version 2.11.0+
The query was compared to classified sequences in ".../1775_20210216_b02.fa-families.fa"
FamDB:

==					
Psi_GCF_017051935.1					
sequences	29				
total length	4773208 b	(4773208 bp excl N/X-runs)			
GC level:	53,74%				
bases mas	29714 bp (	0.62 %)			
==					
	number of elements*	length occupied	percentage of sequence		
--					
Retroeleme	0	0 bp	0,00%		
	SINEs:	0	0 bp	0,00%	
	Penelope	0	0 bp	0,00%	
	LINEs:	0	0 bp	0,00%	
	CRE/SLAC	0	0 bp	0,00%	
	L2/CR1/Re	0	0 bp	0,00%	
	R1/LOA/Jo	0	0 bp	0,00%	
	R2/R4/NeS	0	0 bp	0,00%	
	RTE/Bov-B	0	0 bp	0,00%	
	L1/CIN4	0	0 bp	0,00%	
	LTR eleme	0	0 bp	0,00%	
	BEL/Pao	0	0 bp	0,00%	
	Ty1/Copia	0	0 bp	0,00%	
	Gypsy/DIR	0	0 bp	0,00%	
	Retroviral	0	0 bp	0,00%	
DNA trans	0	0 bp	0,00%		
	hobo-Activ	0	0 bp	0,00%	
	Tc1-IS630-	0	0 bp	0,00%	
	En-Spm	0	0 bp	0,00%	
	MuDR-IS9	0	0 bp	0,00%	
	PiggyBac	0	0 bp	0,00%	
	Tourist/Har	0	0 bp	0,00%	
	Other (Mirage		0	0 bp	0,00
	P-element	Transib)			
Rolling-circ	0	0 bp	0,00%		
Unclassifie	81	21414 bp	0,45%		
Total inters	21414 bp	0,45%			
Small RNA	21	3058 bp	0,06%		

Satellites:	0	0 bp	0,00%
Simple rep	114	4837 bp	0,10%
Low compl	9	405 bp	0,01%

==

* most repeats fragmented by insertions or deletions
have been counted as one element

RepeatMas default mode

run with rmblastn version 2.11.0+
The query was compared to classified sequences in ".../1775_20210216_b02.fa-families.fa"
FamDB:

==
Psi_GCF_017051945.1
sequences 36
total length 4797428 b| (4797428 bp excl N/X-runs)
GC level: 53,80%
bases mas 33826 bp (0.71 %)
==

	number of elements*	length occupied	percentage of sequence	
--				
Retroeleme	0	0 bp	0,00%	
SINEs:	0	0 bp	0,00%	
Penelope	0	0 bp	0,00%	
LINEs:	0	0 bp	0,00%	
CRE/SLAC	0	0 bp	0,00%	
L2/CR1/Re	0	0 bp	0,00%	
R1/LOA/Jo	0	0 bp	0,00%	
R2/R4/NeS	0	0 bp	0,00%	
RTE/Bov-B	0	0 bp	0,00%	
L1/CIN4	0	0 bp	0,00%	
LTR eleme	0	0 bp	0,00%	
BEL/Pao	0	0 bp	0,00%	
Ty1/Copia	0	0 bp	0,00%	
Gypsy/DIR	0	0 bp	0,00%	
Retroviral	0	0 bp	0,00%	
DNA trans	0	0 bp	0,00%	
hobo-Activ	0	0 bp	0,00%	
Tc1-IS630-	0	0 bp	0,00%	
En-Spm	0	0 bp	0,00%	
MuDR-IS9(	0	0 bp	0,00%	
PiggyBac	0	0 bp	0,00%	
Tourist/Har	0	0 bp	0,00%	
Other (Mirage P-element Transib)	0	0 bp	0,00	
Rolling-circ	0	0 bp	0,00%	
Unclassifie	84	25828 bp	0,54%	
Total inters	25828 bp	0,54%		
Small RNA	20	3070 bp	0,06%	

Satellites:	0	0 bp	0,00%
Simple rep	109	4427 bp	0,09%
Low compl	11	501 bp	0,01%

==

* most repeats fragmented by insertions or deletions
  have been counted as one element

RepeatMas default mode

run with rmblastn version 2.11.0+
The query was compared to classified sequences in ".../1775_20210216_b02.fa-families.fa"
FamDB:

==
Psi_GCF_017051975.1
sequences 36
total length 4938266 b| (4938266 bp excl N/X-runs)
GC level: 53,67%
bases mas 36865 bp (0.75 %)
==

	number of elements*	length occupied	percentage of sequence		
Retroeleme	0	0 bp	0,00%		
	SINEs:	0	0 bp	0,00%	
	Penelope	0	0 bp	0,00%	
	LINEs:	0	0 bp	0,00%	
	CRE/SLAC	0	0 bp	0,00%	
	L2/CR1/Re	0	0 bp	0,00%	
	R1/LOA/Jo	0	0 bp	0,00%	
	R2/R4/NeS	0	0 bp	0,00%	
	RTE/Bov-B	0	0 bp	0,00%	
	L1/CIN4	0	0 bp	0,00%	
	LTR eleme	0	0 bp	0,00%	
	BEL/Pao	0	0 bp	0,00%	
	Ty1/Copia	0	0 bp	0,00%	
	Gypsy/DIR	0	0 bp	0,00%	
	Retroviral	0	0 bp	0,00%	
DNA trans|	0	0 bp	0,00%		
	hobo-Activ	0	0 bp	0,00%	
	Tc1-IS630-	0	0 bp	0,00%	
	En-Spm	0	0 bp	0,00%	
	MuDR-IS9(	0	0 bp	0,00%	
	PiggyBac	0	0 bp	0,00%	
	Tourist/Har	0	0 bp	0,00%	
	Other (Mirage P-element Transib)	0	0 bp	0,00	
Rolling-circ	0	0 bp	0,00%		
Unclassifie	87	27592 bp	0,56%		
Total inters	27592 bp	0,56%			
Small RNA	21	3118 bp	0,06%		

Satellites:	0	0 bp	0,00%
Simple rep	127	5706 bp	0,12%
Low compl	9	449 bp	0,01%

==

* most repeats fragmented by insertions or deletions
have been counted as one element

RepeatMas default mode

run with rmblastn version 2.11.0+
The query was compared to classified sequences in ".../1775_20210216_b02.fa-families.fa"
FamDB:

==
Psi_GCF_017052015.1
sequences 36
total length 4938506 b| (4938506 bp excl N/X-runs)
GC level: 53,67%
bases mas 36809 bp (0.75 %)
==

	number of elements*	length occupied	percentage of sequence		
Retroeleme	0	0 bp	0,00%		
	SINEs:	0	0 bp	0,00%	
	Penelope	0	0 bp	0,00%	
	LINEs:	0	0 bp	0,00%	
	CRE/SLAC	0	0 bp	0,00%	
	L2/CR1/Re	0	0 bp	0,00%	
	R1/LOA/Jo	0	0 bp	0,00%	
	R2/R4/NeS	0	0 bp	0,00%	
	RTE/Bov-B	0	0 bp	0,00%	
	L1/CIN4	0	0 bp	0,00%	
	LTR eleme	0	0 bp	0,00%	
	BEL/Pao	0	0 bp	0,00%	
	Ty1/Copia	0	0 bp	0,00%	
	Gypsy/DIR	0	0 bp	0,00%	
	Retroviral	0	0 bp	0,00%	
DNA transp	0	0 bp	0,00%		
	hobo-Activ	0	0 bp	0,00%	
	Tc1-IS630-	0	0 bp	0,00%	
	En-Spm	0	0 bp	0,00%	
	MuDR-IS90	0	0 bp	0,00%	
	PiggyBac	0	0 bp	0,00%	
	Tourist/Har	0	0 bp	0,00%	
	Other (Mirage P-element Transib)		0	0 bp	0,00
Rolling-circ	0	0 bp	0,00%		
Unclassifie	88	27641 bp	0,56%		
Total inters	27641 bp	0,56%			
Small RNA	19	2977 bp	0,06%		

Satellites:	0	0 bp	0,00%
Simple rep	128	5742 bp	0,12%
Low compl	9	449 bp	0,01%

==

* most repeats fragmented by insertions or deletions
have been counted as one element

RepeatMas default mode

run with rmblastn version 2.11.0+
The query was compared to classified sequences in ".../1775_20210216_b02.fa-families.fa"
FamDB:

==						
Psi_GCF_017052095.1						
sequences	37					
total length	5042668 b	(5042668 bp excl N/X-runs)				
GC level:	53,53%					
bases mas	37365 bp (	0.74 %)				
==						
	number of elements*	length occupied	percentage of sequence			
--						
Retroeleme	0	0 bp	0,00%			
	SINEs:	0	0 bp	0,00%		
	Penelope	0	0 bp	0,00%		
	LINEs:	0	0 bp	0,00%		
	CRE/SLAC	0	0 bp	0,00%		
	L2/CR1/Re	0	0 bp	0,00%		
	R1/LOA/Jo	0	0 bp	0,00%		
	R2/R4/NeS	0	0 bp	0,00%		
	RTE/Bov-B	0	0 bp	0,00%		
	L1/CIN4	0	0 bp	0,00%		
	LTR eleme	0	0 bp	0,00%		
	BEL/Pao	0	0 bp	0,00%		
	Ty1/Copia	0	0 bp	0,00%		
	Gypsy/DIR	0	0 bp	0,00%		
	Retroviral	0	0 bp	0,00%		
DNA trans	0	0 bp	0,00%			
	hobo-Activ	0	0 bp	0,00%		
	Tc1-IS630-	0	0 bp	0,00%		
	En-Spm	0	0 bp	0,00%		
	MuDR-IS9	0	0 bp	0,00%		
	PiggyBac	0	0 bp	0,00%		
	Tourist/Har	0	0 bp	0,00%		
	Other (Mirage		0	0 bp		0,00
	P-element	Transib)				
Rolling-circ	0	0 bp	0,00%			
Unclassifie	87	28171 bp	0,56%			
Total inters	28171 bp	0,56%				
Small RNA	21	3014 bp	0,06%			

Satellites:	0	0 bp	0,00%
Simple rep	123	5659 bp	0,11%
Low compl	11	521 bp	0,01%

==

* most repeats fragmented by insertions or deletions
have been counted as one element

RepeatMas default mode

run with rmblastn version 2.11.0+
The query was compared to classified sequences in ".../1775_20210216_b02.fa-families.fa"
FamDB:

==
Psi_GCF_017052115.1
sequences 37
total length 5090281 bp (5090281 bp excl N/X-runs)
GC level: 53,53%
bases mas 37979 bp (0.75 %)
==

	number of elements*	length occupied	percentage of sequence

Retroeleme	0	0 bp	0,00%	
	SINEs:	0	0 bp	0,00%
	Penelope	0	0 bp	0,00%
	LINEs:	0	0 bp	0,00%
	CRE/SLAC	0	0 bp	0,00%
	L2/CR1/Re	0	0 bp	0,00%
	R1/LOA/Jo	0	0 bp	0,00%
	R2/R4/NeS	0	0 bp	0,00%
	RTE/Bov-B	0	0 bp	0,00%
	L1/CIN4	0	0 bp	0,00%
	LTR eleme	0	0 bp	0,00%
	BEL/Pao	0	0 bp	0,00%
	Ty1/Copia	0	0 bp	0,00%
	Gypsy/DIR	0	0 bp	0,00%
	Retroviral	0	0 bp	0,00%
DNA transp	0	0 bp	0,00%	
	hobo-Activ	0	0 bp	0,00%
	Tc1-IS630-	0	0 bp	0,00%
	En-Spm	0	0 bp	0,00%
	MuDR-IS90	0	0 bp	0,00%
	PiggyBac	0	0 bp	0,00%
	Tourist/Har	0	0 bp	0,00%
	Other (Mirage P-element Transib)	0	0 bp	0,00
Rolling-circ	0	0 bp	0,00%	
Unclassifie	87	28183 bp	0,55%	
Total inters	28183 bp	0,55%		
Small RNA	21	3014 bp	0,06%	

Satellites:	0	0 bp	0,00%
Simple rep	135	6261 bp	0,12%
Low compl	11	521 bp	0,01%

==

* most repeats fragmented by insertions or deletions
have been counted as one element

RepeatMas default mode

run with rmblastn version 2.11.0+
The query was compared to classified sequences in ".../1775_20210216_b02.fa-families.fa"
FamDB:

==
Psi_GCF_017052135.1
sequences 23
total length 4673200 b| (4673200 bp excl N/X-runs)
GC level: 53,67%
bases mas 31924 bp (0.68 %)
==

		number of elements*	length occupied	percentage of sequence
Retroeleme		0	0 bp	0,00%
	SINEs:	0	0 bp	0,00%
	Penelope	0	0 bp	0,00%
	LINEs:	0	0 bp	0,00%
	CRE/SLAC	0	0 bp	0,00%
	L2/CR1/Re	0	0 bp	0,00%
	R1/LOA/Jo	0	0 bp	0,00%
	R2/R4/NeS	0	0 bp	0,00%
	RTE/Bov-B	0	0 bp	0,00%
	L1/CIN4	0	0 bp	0,00%
	LTR eleme	0	0 bp	0,00%
	BEL/Pao	0	0 bp	0,00%
	Ty1/Copia	0	0 bp	0,00%
	Gypsy/DIR	0	0 bp	0,00%
	Retroviral	0	0 bp	0,00%
DNA trans|		1	156 bp	0,00%
	hobo-Activ	0	0 bp	0,00%
	Tc1-IS630-	1	156 bp	0,00%
	En-Spm	0	0 bp	0,00%
	MuDR-IS9(	0	0 bp	0,00%
	PiggyBac	0	0 bp	0,00%
	Tourist/Har	0	0 bp	0,00%
	Other (Mirage P-element Transib)	0	0 bp	0,00
Rolling-circ		0	0 bp	0,00%
Unclassifie		86	23254 bp	0,50%
Total inters		23410 bp	0,50%	
Small RNA		22	3136 bp	0,07%

Satellites:	0	0 bp	0,00%
Simple rep	120	5013 bp	0,11%
Low compl	7	365 bp	0,01%

==

* most repeats fragmented by insertions or deletions
have been counted as one element

RepeatMas default mode

run with rmblastn version 2.11.0+
The query was compared to classified sequences in ".../1775_20210216_b02.fa-families.fa"
FamDB:

==

Psi_GCF_017052175.1

sequences 26

total length 4633154 b| (4633154 bp excl N/X-runs)

GC level: 53,71%

bases mas 29013 bp (0.63 %)

==

		number of elements*	length occupied	percentage of sequence
Retroeleme		0	0 bp	0,00%
	SINEs:	0	0 bp	0,00%
	Penelope	0	0 bp	0,00%
	LINEs:	0	0 bp	0,00%
	CRE/SLAC	0	0 bp	0,00%
	L2/CR1/Re	0	0 bp	0,00%
	R1/LOA/Jo	0	0 bp	0,00%
	R2/R4/NeS	0	0 bp	0,00%
	RTE/Bov-E	0	0 bp	0,00%
	L1/CIN4	0	0 bp	0,00%
	LTR eleme	0	0 bp	0,00%
	BEL/Pao	0	0 bp	0,00%
	Ty1/Copia	0	0 bp	0,00%
	Gypsy/DIR	0	0 bp	0,00%
	Retroviral	0	0 bp	0,00%
DNA transp		0	0 bp	0,00%
	hobo-Activ	0	0 bp	0,00%
	Tc1-IS630-	0	0 bp	0,00%
	En-Spm	0	0 bp	0,00%
	MuDR-IS9(	0	0 bp	0,00%
	PiggyBac	0	0 bp	0,00%
	Tourist/Har	0	0 bp	0,00%
	Other (Mirage P-element Transib)	0	0 bp	0,00
Rolling-circ		0	0 bp	0,00%
Unclassifie		82	21183 bp	0,46%
Total inters			21183 bp	0,46%
Small RNA		21	2736 bp	0,06%

Satellites:	0	0 bp	0,00%
Simple rep	113	4801 bp	0,10%
Low compl	6	293 bp	0,01%

==

* most repeats fragmented by insertions or deletions
have been counted as one element

RepeatMas default mode

run with rmblastn version 2.11.0+
The query was compared to classified sequences in ".../1775_20210216_b02.fa-families.fa"
FamDB:

==
Psi_GCF_017052195.1
sequences 24
total length 4673210 b| (4673210 bp excl N/X-runs)
GC level: 53,67%
bases mas 31905 bp (0.68 %)
==

	number of elements*	length occupied	percentage of sequence
Retroeleme	0	0 bp	0,00%
SINEs:	0	0 bp	0,00%
Penelope	0	0 bp	0,00%
LINEs:	0	0 bp	0,00%
CRE/SLAC	0	0 bp	0,00%
L2/CR1/Re	0	0 bp	0,00%
R1/LOA/Jo	0	0 bp	0,00%
R2/R4/NeS	0	0 bp	0,00%
RTE/Bov-E	0	0 bp	0,00%
L1/CIN4	0	0 bp	0,00%
LTR eleme	0	0 bp	0,00%
BEL/Pao	0	0 bp	0,00%
Ty1/Copia	0	0 bp	0,00%
Gypsy/DIR	0	0 bp	0,00%
Retroviral	0	0 bp	0,00%
DNA trans|	1	156 bp	0,00%
hobo-Activ	0	0 bp	0,00%
Tc1-IS630-	1	156 bp	0,00%
En-Spm	0	0 bp	0,00%
MuDR-IS9(	0	0 bp	0,00%
PiggyBac	0	0 bp	0,00%
Tourist/Har	0	0 bp	0,00%
Other (Mirage P-element Transib)	0	0 bp	0,00
Rolling-circ	0	0 bp	0,00%
Unclassifie	86	23235 bp	0,50%
Total inters	23391 bp	0,50%	
Small RNA	22	3136 bp	0,07%

Satellites:	0	0 bp	0,00%
Simple rep	120	5013 bp	0,11%
Low compl	7	365 bp	0,01%

==

* most repeats fragmented by insertions or deletions
have been counted as one element

RepeatMas default mode

run with rmblastn version 2.11.0+
The query was compared to classified sequences in ".../1775_20210216_b02.fa-families.fa"
FamDB:

==
Psi_GCF_017052375.1
sequences 24
total length 4672170 b| (4672170 bp excl N/X-runs)
GC level: 53,67%
bases mas 31978 bp (0.68 %)
==

		number of elements*	length occupied	percentage of sequence	
Retroeleme		0	0 bp	0,00%	
	SINEs:	0	0 bp	0,00%	
	Penelope	0	0 bp	0,00%	
	LINEs:	0	0 bp	0,00%	
	CRE/SLAC	0	0 bp	0,00%	
	L2/CR1/Re	0	0 bp	0,00%	
	R1/LOA/Jo	0	0 bp	0,00%	
	R2/R4/NeS	0	0 bp	0,00%	
	RTE/Bov-B	0	0 bp	0,00%	
	L1/CIN4	0	0 bp	0,00%	
	LTR eleme	0	0 bp	0,00%	
	BEL/Pao	0	0 bp	0,00%	
	Ty1/Copia	0	0 bp	0,00%	
	Gypsy/DIR	0	0 bp	0,00%	
	Retroviral	0	0 bp	0,00%	
DNA transp		1	156 bp	0,00%	
	hobo-Activ	0	0 bp	0,00%	
	Tc1-IS630-	1	156 bp	0,00%	
	En-Spm	0	0 bp	0,00%	
	MuDR-IS90	0	0 bp	0,00%	
	PiggyBac	0	0 bp	0,00%	
	Tourist/Har	0	0 bp	0,00%	
	Other (Mirage P-element Transib)	0	0 bp	0,00	
Rolling-circ		0	0 bp	0,00%	
Unclassifie		86	23254 bp	0,50%	
Total inters	23410 bp	0,50%			
Small RNA		23	3190 bp	0,07%	

Satellites:	0	0 bp	0,00%
Simple rep	120	5013 bp	0,11%
Low compl	7	365 bp	0,01%

==

* most repeats fragmented by insertions or deletions
have been counted as one element

RepeatMas default mode

run with rmblastn version 2.11.0+
The query was compared to classified sequences in ".../1775_20210216_b02.fa-families.fa"
FamDB:
