# Supplementary material for: Analysis of Italian isolates of Pantoea stewartii subsp. stewartii and development of a real-time PCR-based diagnostic method
Source: Front Microbiol. 2023 Apr 27;14:1129229. doi: 10.3389/fmicb.2023.1129229 (PMC10174441; doi:10.3389/fmicb.2023.1129229)
Supplement: Supplementary file 2 [file Data_Sheet_2.docx]

Supplementary Material

Analysis of Italian isolates of Pantoea stewartii subsp. stewartii and development of a real-time PCR based diagnostic method

**Valeria Scala1*†, Luigi Faino2†, Francesca Costantini1, Alessio Albanese2, Nicoletta Pucci1, Massimo Reverberi2, Stefania Loreti1**

*** Correspondence:** Valeria Scala: valeria.scala@crea.gov.it

# Supplementary Figures

**Supplementary Figure 1**. Unrooted phylogenetic tree of all sequenced strains with replicates. The strain GCF_002082215.1 was used as reference for the alignment in RealPhy. RAxML was run with 1000 bootstrap replicates.


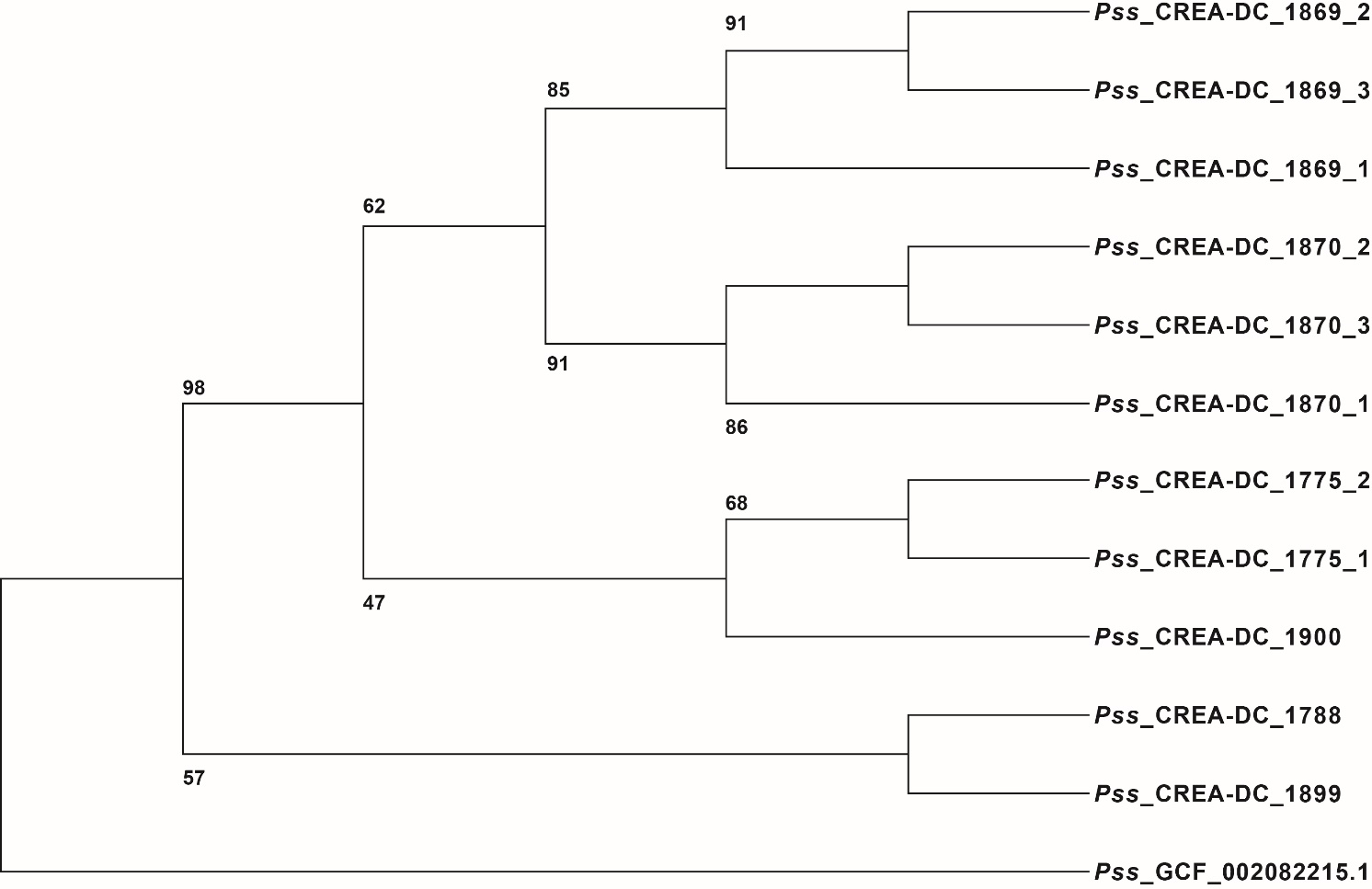


**Supplementary Figure 2**. Region amplified by the primer set ctg3 selected for RT-PCR. In the top is reported the genomic region where the amplicon (blue bar) is located. *Pss*_CREA-DC1869_2 and *Psi*_GCF_017051805.1 were selected as representative of *Pss* and *Psi* strains, respectively. *Pa*_GCF_000233595.1 was selected as representative of *P. agglomerans.* The gray arrowed boxes represent reads from different samples aligned to the reference genome of *Pss*_GCF_002082215.1. Colored boxes within reads show single polymorphic nucleotides in the alignments between the reads and the reference genome


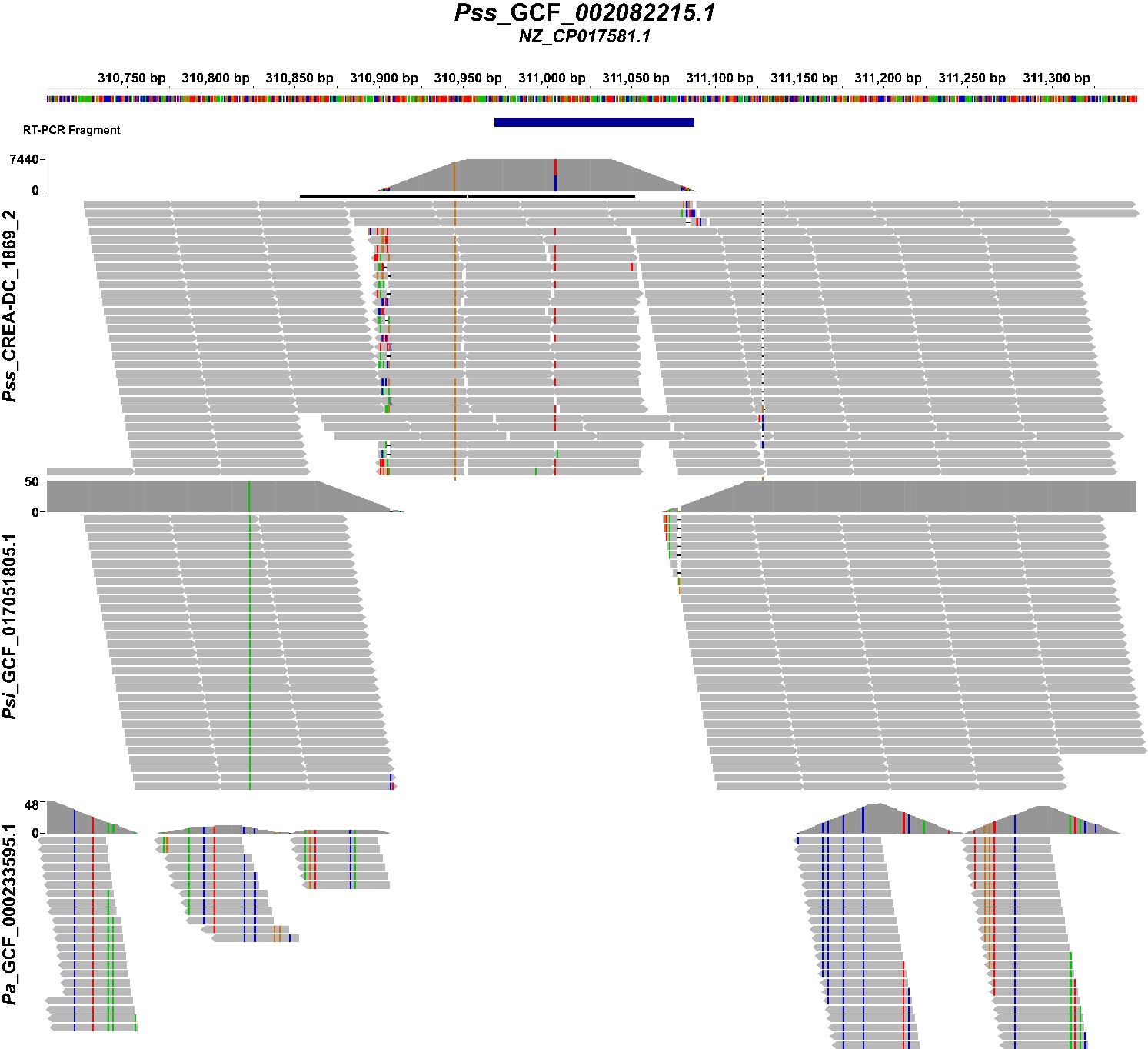


**Supplementary Figure 3. a)** Real-time PCR amplification curves of standard curve employing ctg3 real-time PCR and the genomic DNA of Pss (IPV-BO 2766) 10-fold dilution from 10 ng to 10 fg (type 1 of samples). **b**) Real-time PCR amplification curves of standard curve performed with ctg3 real-time PCR and the genomic DNA extract from 10-fold dilution (from 10^8^ to 10^1^ CFU/ml) of bacterial cells of Pss (IPV-BO 2766) (type 2 of samples. **c)** Real-time PCR amplification curves of the standard curve performed with ctg3 real-time PCR, and the spiked samples (type 3 samples).

**a**


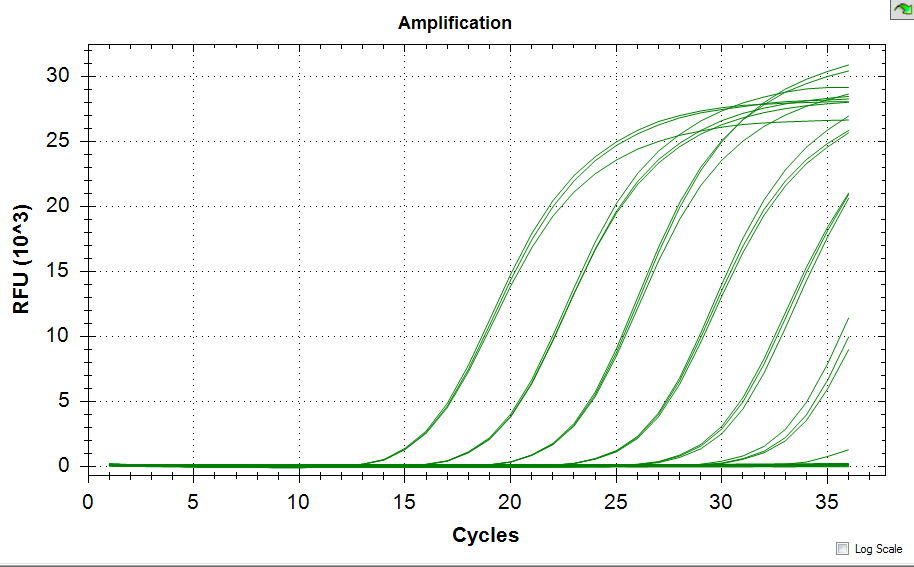


**b**


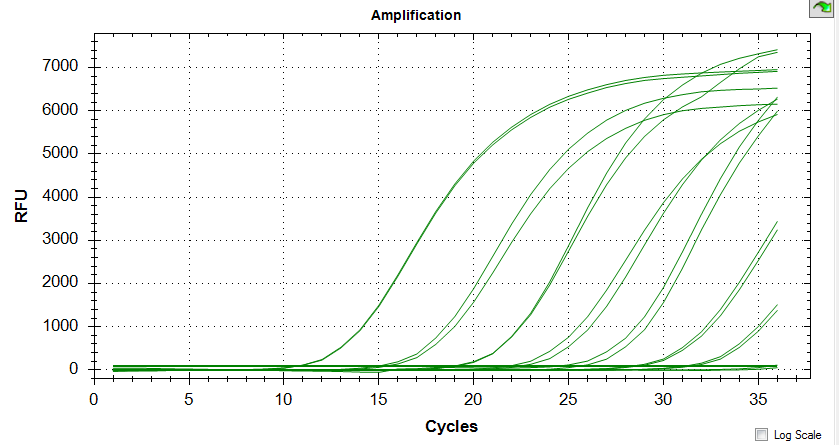


**c**

**
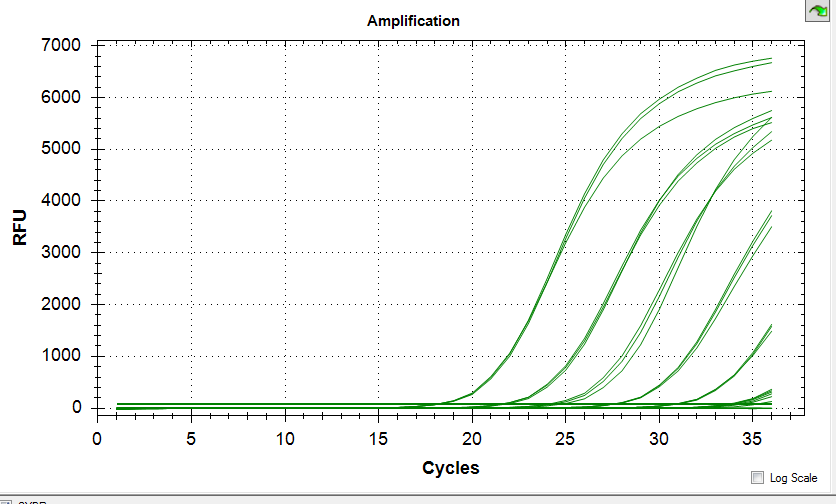
**

**Supplementary Figure 4.** Real-time PCR amplification curves of the ctg3 real-time PCR analytical specificity assay(exclusivity). The figure reports the exclusivity assay performed for *P. agglomerans*, *P. ananatis*, Psi, for all the *Pantoea* spp., for the isolates of sweet maize endophytes and for all the non-*Pantoea* isolates (Ct = N/A). Pss (IPV-BO 2766) was amplified as positive amplification control.


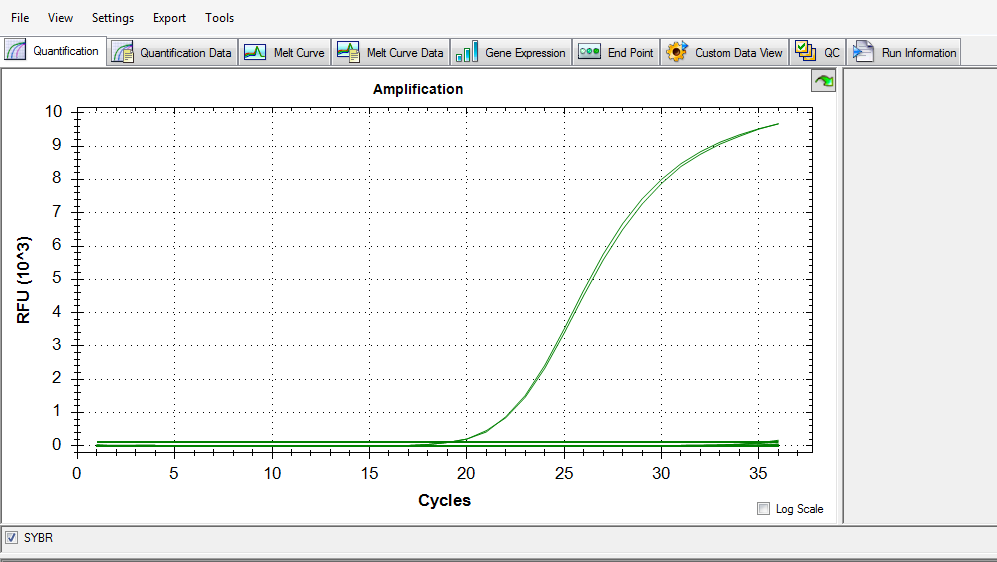


# Supplementary Tables

**Supplementary Table 1.** Repetitive element classification of *Pantoea stewartia* subsp. *Stewartia* CREA-DC 1870 performed using TETools.

| Repetitive element type | | | # elements | Length occupied | % of sequence |
| --- | --- | --- | --- | --- | --- |
| Retroelements |  |  | 0 | 0 | 0 |
|  | SINEs: |  | 0 | 0 | 0 |
|  | Penelope |  | 0 | 0 | 0 |
|  | LINEs: |  | 0 | 0 | 0 |
|  |  | CRE/SLACS | 0 | 0 | 0 |
|  |  | L2/CR1/Rex | 0 | 0 | 0 |
|  |  | R1/LOA/Jockey | 0 | 0 | 0 |
|  |  | R2/R4/NeSL | 0 | 0 | 0 |
|  |  | RTE/Bov-B | 0 | 0 | 0 |
|  |  | L1/CIN4 | 0 | 0 | 0 |
|  | LTR elements: |  | 0 | 0 | 0 |
|  |  | BEL/Pao | 0 | 0 | 0 |
|  |  | Ty1/Copia | 0 | 0 | 0 |
|  |  | Gypsy/DIRS1 | 0 | 0 | 0 |
|  |  | Retroviral | 0 | 0 | 0 |
|  |  |  |  |  |  |
| DNA | transposons |  | 73 | 69499 | 1.26 |
|  | hobo-Activator |  | 0 | 0 | 0 |
|  | Tc1-IS630-Pogo |  | 73 | 69499 | 1.26 |
|  | En-Spm |  | 0 | 0 | 0 |
|  | MuDR-IS905 |  | 0 | 0 | 0 |
|  | PiggyBac |  | 0 | 0 | 0 |
|  | Tourist/Harbinger | | 0 | 0 | 0 |
|  | Other | (Mirage, P-elements,  Transib) | 0 | 0 | 0 |
| Rolling-circles |  |  | 0 | 0 | 0 |
| Unclassified: |  |  | 352 | 300261 | 5.44 |
| Total interspersed repeats | |  |  | 369760 | 6.7 |
| Small RNA |  |  | 32 | 17862 | 0.32 |
| Satellites: |  |  | 0 | 0 | 0 |
| Simple | repeats: |  | 133 | 5984 | 0.11 |
| Low | complexity: |  | 6 | 245 | 0 |
